# DNA-PK participates in pre-rRNA biogenesis independent of DNA double-strand break repair

**DOI:** 10.1093/nar/gkae316

**Published:** 2024-04-29

**Authors:** Peng Li, Xiaochen Gai, Qilin Li, Qianqian Yang, Xiaochun Yu

**Affiliations:** Westlake Laboratory of Life Sciences and Biomedicine, Hangzhou, Zhejiang, China; School of Life Sciences, Westlake University, Hangzhou, Zhejiang, China; Institute of Basic Medical Sciences, Westlake Institute for Advanced Study, Hangzhou, Zhejiang, China; Westlake Laboratory of Life Sciences and Biomedicine, Hangzhou, Zhejiang, China; School of Life Sciences, Westlake University, Hangzhou, Zhejiang, China; Institute of Basic Medical Sciences, Westlake Institute for Advanced Study, Hangzhou, Zhejiang, China; Westlake Laboratory of Life Sciences and Biomedicine, Hangzhou, Zhejiang, China; School of Life Sciences, Westlake University, Hangzhou, Zhejiang, China; Institute of Basic Medical Sciences, Westlake Institute for Advanced Study, Hangzhou, Zhejiang, China; Westlake Laboratory of Life Sciences and Biomedicine, Hangzhou, Zhejiang, China; School of Life Sciences, Westlake University, Hangzhou, Zhejiang, China; Institute of Basic Medical Sciences, Westlake Institute for Advanced Study, Hangzhou, Zhejiang, China; Westlake Laboratory of Life Sciences and Biomedicine, Hangzhou, Zhejiang, China; School of Life Sciences, Westlake University, Hangzhou, Zhejiang, China; Institute of Basic Medical Sciences, Westlake Institute for Advanced Study, Hangzhou, Zhejiang, China

## Abstract

Although DNA-PK inhibitors (DNA-PK-i) have been applied in clinical trials for cancer treatment, the biomarkers and mechanism of action of DNA-PK-i in tumor cell suppression remain unclear. Here, we observed that a low dose of DNA-PK-i and PARP inhibitor (PARP-i) synthetically suppresses *BRCA*-deficient tumor cells without inducing DNA double-strand breaks (DSBs). Instead, we found that a fraction of DNA-PK localized inside of nucleoli, where we did not observe obvious DSBs. Moreover, the Ku proteins recognize pre-rRNA that facilitates DNA-PKcs autophosphorylation independent of DNA damage. Ribosomal proteins are also phosphorylated by DNA-PK, which regulates pre-rRNA biogenesis. In addition, DNA-PK-i acts together with PARP-i to suppress pre-rRNA biogenesis and tumor cell growth. Collectively, our studies reveal a DNA damage repair-independent role of DNA-PK-i in tumor suppression.

## Graphical Abstract

**Graphical Abstract:**
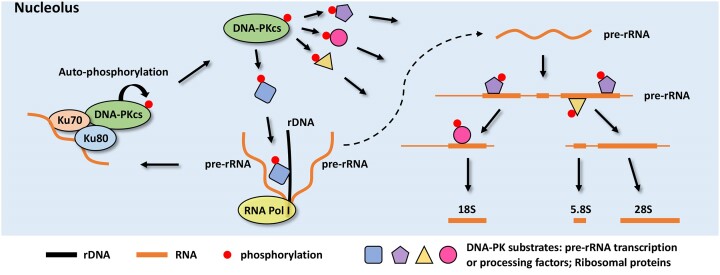

## Introduction

DNA-dependent protein kinase (DNA-PK) plays a key role in classical non-homologous end joining (cNHEJ), one of the major pathways that repairs DNA double-strand breaks (DSBs) in mammal cells (1). DNA-PK is a protein complex composed of the DNA-dependent protein kinase catalytic subunit (DNA-PKcs) and a heterodimer of Ku proteins (aka the Ku70/80 heterodimer) (2). In response to DSBs, cNHEJ is conducted in four distinct steps: (a) detection of the DSB ends by the Ku70/80 heterodimer; (b) recruitment of DNA-PKcs to tether the DSB ends together and protect them from nucleolytic digestion; (c) removal of non-ligatable ends by Artemis and (d) ligation of the DSB ends by Ligase 4 (LIG4) (3).

A considerable number of studies have demonstrated the association between dysregulated DNA-PK and cancer development (4). Overexpression of DNA-PK is frequently found in a variety of cancer types and is associated with poor prognostics (5–7). Hence, DNA-PK is considered as a cancer therapeutic target for mono-agent therapy, or in combination with radiotherapy or chemotherapy (8). Numerous highly selective DNA-PK-i, such as M3814 and NU7441, have been developed (9,10) and applied in clinical trials for different types of cancer (NCT03770689, NCT04172532, NCT04533750). Although some promising preclinical and early-phase clinical results have been reported recently (9,11,12), the precise management of DNA-PK-targeted therapy remains a challenge due to the limited knowledge on the physiological functions of DNA-PK and lack of biomarkers that predict the efficacy of DNA-PK inhibitors in cancer treatment.

Among the DNA-PK complex, DNA-PKcs is a phosphatidylinositol-3 kinase-like kinase (PIKK), and is evolutionarily conserved in most eukaryotes. DNA-PKcs phosphorylates a number of substrates. Interestingly, accumulated evidence shows that the substrates of DNA-PKcs are not only limited in DSB repair but also existed during many other cellular processes, such as gene transcription (13,14), tumor cell metastasis (13), mitosis (15), and innate and adaptive immunity (16,17). However, it is unclear how DNA-PKcs is activated in other cellular processes. Previous studies show that the Ku70/80 heterodimer recognizes RNA (18,19). It has been reported that, in yeast, the Ku70/80 heterodimer is able to recognize a 48-nucleotide (nt) hairpin of TLC1, a telomerase RNA, to regulate TLC1 nuclear retention and telomerase accumulation at telomeres (19,20), which protects the chromosome ends from deleterious fusions and degradation (21). Thus, it is possible that the Ku70/80 heterodimer recognizes different nucleic acid species to activate DNA-PKcs during different biological processes.

Although DNA-PK regulates multiple biological processes, mice lacking either DNA-PKcs or Ku are still viable (22–24), indicating that DNA-PK is not essential during prenatal development. Poly(ADP-ribose) polymerase 1 (PARP1), known as another DNA repair factor, is an enzyme responsible for catalyzing poly(ADP-ribosyl)ation (PARylation). PARylation is a unique post-translational modification that plays a critical role in the cellular response to genotoxic stress and serves as an early alarm for sensing DNA damage (25). Similar to the DNA-PK knockout mice, mice lacking PARP1 do not display obvious developmental defects (26–28), suggesting that neither DNA-PK nor PARP1 is required for prenatal development. However, we notice that mice lacking both DNA-PK and PARP1 die during early embryonic development (29), indicating that DNA-PK and PARP1 may act synergistically during the early development stage. Since PARP1 also participates in DNA damage repair, it has been hypothesized that loss of both DNA-PK and PARP1 induce synthetic lethality for tumor cells due to loss of multiple repair pathways. However, when we treated tumor cells with both DNA-PK-i and PARP-i, we did not observe elevated DNA lesions. Interestingly, a recent study also shows that DNA-PK regulates rRNA processing (30). Consistently, we found that DNA-PK facilitates pre-ribosome biogenesis, and inhibition of both DNA-PK and PARPs impairs pre-ribosome biogenesis. Thus, our study may provide a mechanism of action by which DNA-PK-i and PARP-i suppress tumor cell growth.

## Materials and methods

### Materials

These following antibodies were purchased from respective company: anti-Ku70 (CST, 4588; Santa Cruz, sc-1486), anti-Ku80 (CST, 2180; Santa Cruz, sc-1484), anti-DNA-PKcs (CST, 38168; Thermo Fisher, MA5-32192), anti-phospho-DNA-PKcs-T2609 (Thermo Fisher, PA1-29541), anti-FBL (Santa Cruz, sc-374022; CST, 2639), anti-NPM1 (Santa Cruz, sc-271737), anti-RPA194 (Santa Cruz, sc-48385), anti-ATM phospho-S1981 (Rockland, 200-301-400), anti-puromycin (Sigma-Aldrich, MABE343), anti-γH2AX (CST, 9718S), anti-Artemis (GeneTex, HL1227), anti-53BP1 (Abcam, ab172580; Novus, NBP2-25028), anti-phospho-RPS6-S235/236 (CST, 2211), anti-PARP10 (Santa Cruz, sc-53858), anti-GAPDH (CST, 2118). All other reagents including BMH-21 (Selleck, S7718), M3814 (Selleck, S8586), olaparib (Selleck, S1060), rucaparib (Selleck, S4948), talazoparib (Selleck, S7048), and etoposide (Selleck, S1225) were obtained from commercial sources.

### Plasmids and construction

The Ku80 cDNA was subcloned into the SBP-puro vector for transient transfection. DNA-PKcs or Ku70 CRISPR-KO constructs were made according to a previously described protocol (31). The sgRNA target sequences for DNA-PKcs and Ku70 were as following: TTGTCCGCTGCGGACCGCTG, ATGTAGTGCCATTCGGTGTG. The sgRNAs were subcloned into LentiCRISPR v2 vector (Addgene). Besides, the DNA-PKcs or Ku70 cDNA was transferred into the pLenti-Dest vector (Thermo Fisher). Various mutations of DNA-PKcs (T2609A, D3922A) or Ku70 (1–438 truncation) were further introduced using standard mutagenesis techniques, which were confirmed by DNA sequencing analysis. The DNA-PKcs or Ku70 knockout HeLa/HEK293T cell lines, and the DNA-PKcs or Ku70 mutant reconstituted cell lines as indicated were subsequently constructed according to a previously described protocol (32).

### Cell culture

All the cells were cultured according to the directions from ATCC. HEK293T, HeLa, MDA-MB-231 and MDA-MB-436 cells were maintained in the high-glucose DMEM medium supplemented with 10% FBS. HCC1937 and HCC1937-BRCA1 cells (a myc tagged BRCA1 was stably expressed in HCC1937 cells, which was obtained from Dr Junjie Chen's group) (33) were maintained in RPMI1640 medium supplemented with 15% FBS. UWB1.289 (UWB1) cells were maintained in RPMI1640 medium and MEGM bullet kit (1:1; Lonza) with 3% FBS. UWB1.289-SYr12 (SYr12) cells were maintained in RPMI1640 medium and MEGM bullet kit (1:1; Lonza) with 3% FBS and 1 μM PARP-i (olaparib; Selleck).

### Comet assay

The comet assay was conducted according to published procedures (34). In brief, HCC1937 cells were pre-treated with M3814 (1 μM) and/or olaparib (1 μM) for 2 days, and etoposide (1 μM for 24 h) was used as the positive control. Then, the cells were mixed with 0.5% low-melting temperature agarose (Agarose LMP, Gen-view Scientific) at 37°C, placed on a precleaned microscope slide that was already covered with a second layer of 0.5% normal melting agarose (Regular Agarose G-10, Biowest) and immediately covered with a coverglass and kept at 4°C for 5 min. After the coverglass was gently removed, the slide was covered with a third layer of low-melting agarose by using another coverglass, then horizontally placed at 4°C. The solidified slide was then immersed in a lysing solution (1% sodium sarcosinate, 2.5 M NaCl, 100 mM Na_2_-EDTA, 10 mM Tris pH 10.0 and 1% freshly added Triton X-100) for 1 h to release and unfold the DNA, followed by the electrophoretic buffer (1 mM Na_2_-EDTA and 300 mM NaOH) for 20 min to un-wind the DNA. The electrophoresis was then conducted for another 20 min at 25 V. The slide was then horizontally immersed in 0.4 M Tris–HCl (pH 7.5) and stained with DAPI (Sigma-Aldrich) for 5 min before observation under the Zeiss LSM 800 Airyscan inverted confocal microscope. The tail moment of comets was measured using Comet Assay IV software.

### Immunofluorescence microscopy

After the indicated treatment as shown in figure legends, cells were washed once with 1 × PBS, when indicated, incubated with CSK buffer (10 mM Pipes with pH 7.0, 100 mM NaCl, 300 mM sucrose, 3 mM MgCl_2_ and 0.7% Triton X-100) for 3 min at room temperature for pre-extraction, and then, fixed with 4% paraformaldehyde for 20 min at room temperature followed by three times wash with 1 × PBS. The cells were permeabilized with 0.25% Triton X-100 in 1 × PBS for 10 min and blocked with 1 × PBS containing 2% BSA for 1 h. Fixed cells were incubated with primary antibodies at 4°C overnight, followed by the incubation with the fluorescent secondary antibody for 1 h at room temperature (RT). Cells were washed three times with 1 × PBS for 5 min and stained with DAPI (Thermo Fisher Scientific) for 2 min. Cells were washed with 1 × PBS and mounted with the Fluor Save reagent (Millipore). The fluorescence images were then collected with a Zeiss LSM 800 Airyscan inverted confocal microscope or a Structured illumination microscope (SIM). All the images were acquired with the same microscope settings.

### Short interfering RNAs and transfection

To knockdown the expression of Ku70, Ku80, DNA-PKcs, or RPA194, siRNA targeting Ku70, Ku80, DNA-PKcs or RPA194 was purchased from RiboBio Co. Sequences for siRNA oligo are listed as following:

Ku70-siRNA1:

Sense: 5′-CCAAGUGAGCAGUAACCAATT-3′

Antisense: 5′-UUGGUUACUGCUCACUUGGTT-3′

Ku70-siRNA2:

Sense: 5′-GUGAUGUCCAAUUCAAGAUTT-3′

Antisense: 5′-AUCUUGAAUUGGACAUCACTT-3′

Ku70-siRNA3:

Sense: 5′-GCAUCUUCCUUGACUUGAUTT-3′

Antisense: 5′-AUCAAGUCAAGGAAGAUGCTT-3′

Ku80-siRNA1:

Sense: 5′-GCAAAGAAGGUGAUAACCATT-3′

Antisense: 5′-UGGUUAUCACCUUCUUUGCTT-3′

Ku80-siRNA2:

Sense: 5′-GGUUCUCAACAAGCUGACUTT-3′

Antisense: 5′-AGUCAGCUUGUUGAGAACCTT-3′

Ku80-siRNA3:

Sense: 5′-GAGCAGCGCUUUAACAACUTT-3′

Antisense: 5′-AGUUGUUAAAGCGCUGCUCTT-3′

DNA-PKcs-siRNA1:

Sense: 5′-GAUCGCACCUUACUCUGUUTT-3′

Antisense: 5′-AACAGAGUAAGGUGCGAUTT-3′

DNA-PKcs-siRNA2:

Sense: 5′-CUUUAUGGUGGCCAUGGAGTT-3′

Antisense: 5′-CUCCAUGGCCACCAUAAAGTT-3′

DNA-PKcs-siRNA3:

Sense: 5′-CAACAUAGAGCAGUACAUUTT-3′

Antisense: 5′-AAUGUACUGCUCUAUGUUGTT-3′

RPA194-siRNA1:

Sense: 5′-GAGGCAACGUAUCAUUGAA-3′

Antisense: 5′-UUCAAUGAUACGUUGCCUC-3′

RPA194-siRNA2:

Sense: 5′-GACCGAUCCUUUUUGAGUA-3′

Antisense: 5′-UACUCAAAAAGGAUCGGUC-3′

RPA194-siRNA3:

Sense: 5′-GCAAGCUCAUUGCUCUCUU-3′

Antisense: 5′-AAGAGAGCAAUGAGCUUGC-3′

Negative control siRNA:

Sense: 5′-UUCUCCGAACGUGUCACGUTT-3′

Antisense: 5′-ACGUGACACGUUCGGAGAATT-3′

HeLa cells plated in six-well plates were transfected with siRNAs following the protocol of the manufacturer.

### PAR-CLIP assay

After the pre-treatment with 6SG (0.1 mM) for 14 h, HEK293T cells expressing Ku80 protein with SBP and tandem FLAG tags were washed once with 1 × PBS, and then, removed the PBS completely. Put plates on a tray filled with ice to keep cells cold and irradiated the cells uncovered with 0.4 J cm^2^ total energy of 365 nm UV. The cells were washed with cold PBS and collected by centrifugation at 5000 rpm for 5 min at 4°C, and then, were treated with freshly prepared cold NETN300 lysis buffer (50 mM Tris pH 7.5, 300 mM NaCl, 2 mM EDTA, 1% NP40 and 0.5 mM DTT), pipetted to resuspend, and lysis for 5 min on ice. After centrifugation at 12000 rpm for 15 min at 4°C, the supernatant was transferred to a new tube and diluted with NETN0 (50 mM Tris pH 7.5, 2 mM EDTA, 1% NP40 and 0.5 mM DTT) to the final concentration as NETN100 (50 mM Tris pH 7.5, 100 mM NaCl, 2 mM EDTA, 1% NP40 and 0.5 mM DTT). Then, Ku80 with the SBP tag was immunoprecipitated. High Capacity Streptavidin Resin was added into the lysate and incubated for 2 h at 4°C with rotation. After immunoprecipitation, the beads were washed for 3 times with cold NETN300 wash buffer (50 mM Tris pH 7.5, 300 mM NaCl, 0.05% NP40 and 0.5 mM DTT), 2 times with cold NETN100 wash buffer (50 mM Tris pH 7.5, 100 mM NaCl, 0.05% NP40 and 0.5 mM DTT). The interactome was eluted twice with 500 μl biotin for 15 min at 4°C. M2 Flag beads were added into the elution and incubated another 1.5 h at 4°C with rotation. After immunoprecipitation, the beads were washed for 3 times with cold NETN300 wash buffer, and 2 times with cold NETN100 wash buffer. And the coprecipitated RNAs were isolated by TRIzol RNA extraction reagent according to manufacturer's instructions. The RNA sample was further analysis by RNA-seq. The total RNA amount purified from the Ku complex (SFB-Ku80) and empty control (SFB-vector) are compared. The relative amount of purified rRNA is calculated based on the percentage of sequencing reads of rRNA in total RNA and the amount of total RNA.

### RNA immunoprecipitation

HET293T cells were harvested and treated by freshly prepared nuclear isolation buffer (1.28 M sucrose, 40 mM Tris–HCl pH 7.5, 20 mM MgCl_2_, 4% Triton X-100) on ice for 20 min (with frequent mixing). Nuclear pellets were collected by centrifugation at 2500 g for 15 min and were then treated with freshly prepared RIP buffer (150 mM KCl, 25 mM Tris pH 7.4, 5 mM EDTA, 0.5 mM DTT, 0.5% NP40, 100 U/ml RNAase inhibitor, protease inhibitors). Nuclear membrane and debris were pelleted by centrifugation at 13 000 rpm for 10 min at 4°C. Aliquot of lysate were used for reference RNA isolation. Protein A/G beads was added into the lysate and incubated for 1 h at 4°C with rotation (wash bead first according to Invitrogen Manu). Subsequently, the beads were pelleted by magnet stand and discarded. The supernatant was recycled and incubated with antibodies to the proteins of interest overnight at 4°C with rotation. Protein A/G beads were then added and incubated for another 2 h at 4°C with rotation. After immunoprecipitation, the beads were washed for 6 times with RIP buffer, one time with PBS and one time with urea. 5% of the beads were collected for SDS-PAGE analysis. And the coprecipitated RNAs were isolated by TRIzol RNA extraction reagent according to manufacturer's instructions. RT-qPCR was followed to analyze the coprecipitated rRNAs. q-PRC primers were listed as following:

45S:

F: CCCACCCTCGGTGAGAAAAG

R: GGAAGCGGAGGAGGGTCCTC

ITS1:

F: CGAGAGCCGGAGAACTCGG

R: GCCGACACCCACGTCGTC

ITS2:

F: GGTCCGGAAGGGGAAGGGTG

R: GCGGGGCCTCGGAGGAG

7SK:

F: GGGTTGATTCGGCTGATCT

R: CTCCTCTATCGGGGATGGTC

MALAT1:

F: GAGCGAGTGCAATTTGGTGATG

R: ATCCTCTACGCACAACGCC

### Quantitative phosphoproteomic analysis of DNA-PK substrates

HeLa cells were pre-treated with or without DNA-PK-i. Then, 5 mg of total protein was extracted from cells of each condition using RIPA lysis buffer. Protein was reduced in 10 mM dithiothreitol at 55°C for 45 min followed by alkylation in 50 mM iodoacetamide at room temperature in the dark for 30 min. Protein was precipitated using acetone and then resuspended in 100 mM triethylammonium bicarbonate. Trypsin was added at a 1:100 ratio (w/w, enzyme/protein) and the mixture was then incubated at 37°C overnight in the ThermoMixer R with shaking at 1000 rpm. After digestion, peptides were desalted using a HLB cartridge (Waters, WAT094226). Isolation of phosphopeptides was performed as the instruction of the Fe-NTA Phosphopeptide Enrichment Kit (ThermoFisher Scientific, A32992). Enriched phosphopeptides were resuspended in 100 μl of 50 mM triethylammonium bicarbonate and proceed with isobaric peptide labeling with TMT reagents (Thermo Fisher Scientific, 90110). Labeled peptides were combined and desalted using a HLB cartridge (Waters, WAT094225) and the sample was injected onto a Waters XBridge BEH C18 4.6 mm × 250 mm column using a Thermo Dinex Ultimate 3000. Bound peptides were separated using a 40 min gradient. Twenty fractions were collected and concatenated into five total fractions post-run.

Isolated phosphopeptides were resuspended in 20 μl of 0.1% formic acid (w/w) and 6.5 μl were automatically injected using a Thermo EASYnLC 1200 onto a Thermo Acclaim PepMap 0.075 × 20 mm C18 trapping column and washed for approximately 5 min with 0.1% formic acid (w/w). Bound peptides were then eluted over 125 min onto a Thermo Acclaim PepMap 0.075 × 500 mm analytical column at a constant flow rate of 300 nl/min. Eluted peptides were sprayed into a Thermo Scientific Q Exactive HF-X mass spectrometer (www.thermo.com) using a Nanopray ion source.

Data files from the LC–MS/MS analysis were processed using Proteome Discoverer (version 2.5) to identify proteins and calculate isobaric tag intensities using the Sequest HT search engine with default parameters and an FDR of <0.01 at the protein and peptide levels. The *Homo sapiens* proteome obtained from Uniprot was used for the search. Through a comparative analysis of the groups pre-treated with and without DNA-PK-i, a comprehensive quantitative phosphoproteomic investigation revealed a total of 924 DNA-PK substrates, as presented in Supplementary Table S3. Furthermore, Supplementary Table S2 provides detailed information regarding the specific phosphorylation sites on these substrates mediated by DNA-PK.

### Northern blot analysis of pre-rRNA processing

Total RNA was extracted using TRIzol (Life Technology) and 4 μg total RNA was analyzed for each sample. Oligo probes measuring pre-rRNA processing were listed as following:

ITS1: CCTCGCCCTCCGGGCTCCGTTAATGATC

GAPDH: AGGCGCCCAATACGACCAAATCCGTTGACT

### Neosynthesized proteins testing by puromycin incorporation

Neosynthesized proteins were examined as previously described (35). Briefly, HCC1937 and HCC1937-BRCA1 cells were pre-treated with DNA-PK-i (M3814 or NU7441, 1 μM) and/or PARP-i (olaparib, 1 μM) for 24 h. Then, the indicated cells were labeled with puromycin for 30 min. Whole cell extracts were prepared from same number of cells in each condition. Puromycin incorporation was visualized by western blotting with an antibody against puromycin.

### ChIP assays

About 1 × 10^7^ HEK293T cells per 100 mm^3^ culture dish were cross-linked with formaldehyde (final concentration, 0.75%) for 10 min at room temperature (RT). Then the reaction was stopped by adding glycine (final concentration, 125 mM) for 5 min RT. The cells were sonicated in ChIP lysis buffer (50 mM HEPES–KOH pH 7.5, 140 mM NaCl, 1 mM EDTA pH 8, 1% Triton X-100, 0.1% sodium deoxycholate, 0.5% SDS and protease inhibitors) to achieve a chromatin size of ∼300 bp. Fragmented chromatin containing ∼25 μg DNA was immunoprecipitated with 10 μg of specific antibody or control IgG for 1 h at 4°C with rotation. Then, the mixture was incubated with 100 μl ChIP-Grade Protein A/G Magnetic Beads overnight at 4°C with rotation. Immune complexes were washed with the following buffers: low-salt wash buffer (0.1% SDS, 1% Triton X-100, 2 mM EDTA, 20 mM Tris–HCl pH 8.0, 150 mM NaCl), high-salt wash buffer (0.1% SDS, 1% Triton X-100, 2 mM EDTA, 20 mM Tris–HCl pH 8.0, 500 mM NaCl), LiCl wash buffer (0.25 M LiCl, 1% NP-40, 1% sodium deoxycholate, 1 mM EDTA, 10 mM Tris–HCl pH 8) and elution buffer (1% SDS, 100 mM NaHCO_3_). After elution, cross-link reversal, and proteinase K digestion of the immunoprecipitated chromatin, DNA was recovered using QIAquick PCR Purification kit (QIAGEN) and analyzed by qPCR. Primers used for ChIP-qPCR are shown as following:

H0.4-F: 5′-CAGGCGTTCTCGTCTCCG

H0.4-R: 5′-CACCACATCGATCGAAGAGC

H4-F: 5′-CGACGACCCATTCGAACGTCT

H4-R: 5′-CTCTCCGGAATCGAACCCTGA

H8-F: 5′-AGTCGGGTTGCTTGGGAATGC

H8-R: 5′-CCCTTACGGTACTTGTTGACT

H13-F: 5′-ACCTGGCGCTAAACCATTCGT

H13-R: 5′-GGACAAACCCTTGTGTCGAGG

H18-F: 5′-GTTGACGTACAGGGTGGACTG

H18-R: 5′-GGAAGTTGTCTTCACGCCTGA

H33-F: 5′-ATCTCTTGACCTCGTGACCCG

H33-R: 5′-TTGCGTTTCTCTGGACTGACTTC

### Cell proliferation measurement

**Figure 1.**
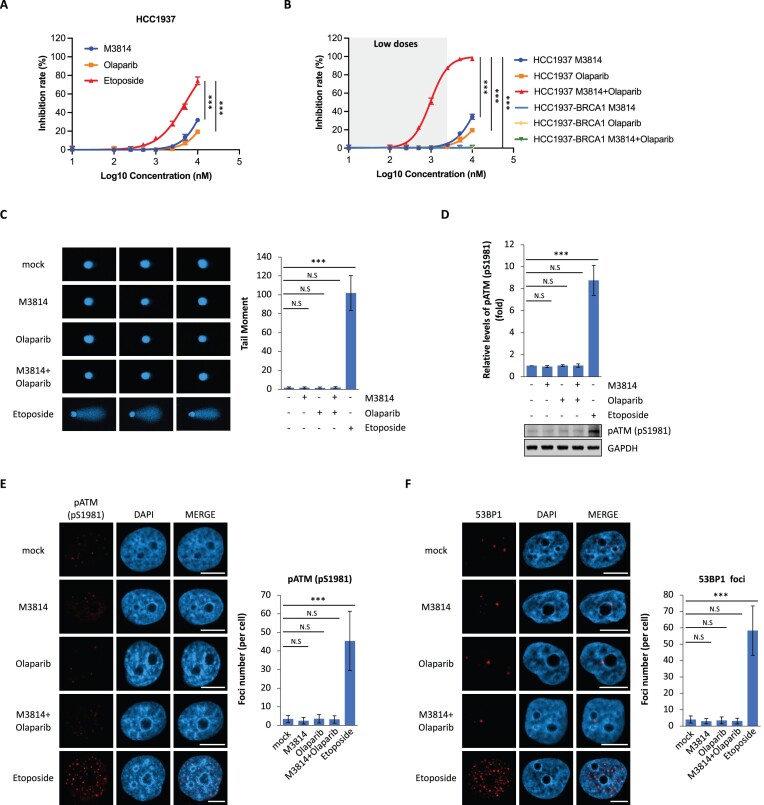
DNA-PK-i and PARP-i act together to suppress *BRCA*-deficient tumor cell growth. (**A**) M3814 or olaparib alone does not suppress the growth of HCC1937 cells *in vitro*. Etoposide treatment was used as a positive control to suppress the growth of HCC1937. HCC1937 cells were treated with indicated dose of M3814, olaparib or etoposide for 7 days. Cell growth was measured with CellTiter-Glo assays. Average cell viability is presented as mean ± SD. ***P < 0.001. (**B**) M3814 and olaparib synergistically suppress the growth of HCC1937 cells. HCC1937 or HCC1937-BRCA1 (a myc-tagged BRCA1 was stably expressed in HCC1937) cells were treated with indicated dose of M3814 and/or olaparib for 7 days. Cell growth was measured with CellTiter-Glo assays. Average cell viability is presented as mean ± SD. ***P < 0.001. (**C**) Low dose of M3814 and/or olaparib treatment do not induce DSBs. HCC1937 cells were pre-treated with M3814 (1 μM) and/or olaparib (1 μM) for 2 days. DSBs were examined using Comet assays. Etoposide (1 μM for 24 h) was used as the positive control. (**D**) Low dose of M3814 and/or olaparib treatment do not activate ATM. HCC1937 cells were pre-treated with M3814 (1 μM) and/or olaparib (1 μM) for 2 days. pSer1981 of ATM was examined by Western blot. Etoposide (1 μM for 24 h) was used as the positive control. (E, F) Low dose of M3814 and/or olaparib treatment do not induce DSB foci formation. HCC1937 cells were pre-treated with M3814 (1 μM) and/or olaparib (1 μM) for 2 days. Foci of pATM (pSer1981) (**E**) or 53BP1 (**F**) was examined by IF. Etoposide (1 μM for 24 h) was used as the positive control. Image bar: 10 μm. ***P < 0.001.

HCC1937 cells, HCC1937-BRCA1 cells, UWB1 cells, UWB1-BRCA1 cells, SYr12 cells, MDA-MB-436 cells or MDA-MB-231 cells were plated into 96-well plates at densities of 1000 cells/well. On the second day, cells were treated as indicated in figure legends as following: in Figure 1A, HCC1937 cells were treated with indicated dose of M3814, olaparib or etoposide for 7 days; in Figure 1B, HCC1937 or HCC1937-BRCA1 cells were treated with indicated dose of M3814 and/or olaparib for 7 days; in Figure 6D, UWB1 cells were treated with the indicated dose of M3814, olaparib, or M3814 together with 10 nM of BMH-21 for 7 days; in Figure 6E, SYr12 cells were treated with the indicated dose of M3814, olaparib, or M3814 together with 10 nM of BMH-21 for 7 days; in Figure 6F, HCC1937 cells were treated with the indicated dose of M3814, olaparib, or M3814 together with 10 nM of BMH-21 for 7 days; in Supplementary Figure S1, HCC1937 cells were treated with the indicated dose of M3814 and/or PARP-i [rucaparib (A) or talazoparib (B)] for 7 days; in Supplementary Figure S2A, UWB1 or UWB1-BRCA1 cells were treated with the indicated dose of M3814 and/or olaparib for 7 days; in Supplementary Figure S2B, MDA-MB-436 or MDA-MB-231 cells were treated with the indicated dose of M3814 and/or olaparib for 7 days; in Supplementary Figure S20A, UWB1 cells were treated with the indicated dose of BMH-21, or BMH-21 together with 100 nM M3814 for 7 days; in Supplementary Figure S20B, SYr12 cells were treated with the indicated dose of BMH-21, or BMH-21 together with 100 nM M3814 together for 7 days. Cell viability was measured using the CellTiter-Glo assays (Promega) according to the manufacturer's instructions. Briefly, after incubation, room temperature CellTiter-Glo reagent was added 1:1 to each well, and the plates were incubated at room temperature for 2 min. Luminescence was measured with the Synergy HT Multi-Detection Microplate Reader and was normalized against control cells.

### Statistical analysis

Data were analyzed with Graphpad Prism 8.0 software. Data were derived from the average of three biological replicate experiments and were presented as the mean ± SD. Statistical significance between two groups was subjected to Student's two-tailed *t* test. *P*< 0.001 was considered statistically significant.

## Results

### DNA-PKcs-i and PARP-i acts together to suppress *BRCA*-deficient tumor cell growth

DNA-PK-i has been used in clinical trials for different types of cancers (9,11,12). However, the selective biomarkers for cancer patients to be treated with DNA-PK-i are still unavailable. There are three major types of repair mechanisms for DSBs, namely NHEJ, homologous recombination (HR) and microhomology-mediated end joining (MMEJ). It has been shown that DNA-PK, PARP1 and BRCA1/2 governs these three repair pathways (36). Although PARP-i has been applied for cancer patients with *BRCA* mutations, >50% tumor patients with *BRCA* mutations do not respond well to PARP-i (37,38). Thus, we ask if combination of DNA-PK-i and PARP-i is able to synergistically kill tumor cells with *BRCA* mutations, when all three DSB repair pathways are suppressed. We chose HCC1937 cells, a triple-negative breast cancer cell line lacking BRCA1, but not sensitive to any known PARP-i (32,39). We found that low dose of either DNA-PK-i (M3814) or PARP-i (olaparib) treatment alone could not suppress the growth of HCC1937 (Figure 1A). However, combination treatment of very low dose of DNA-PK-i and PARP-i remarkably impaired the growth of HCC1937 (Figure 1B). This result was validated by combination treatment of DNA-PK-i with two other PARP-i, rucaparib and talazoparib (Supplementary Figure S1). In contrast, this combination treatment did not affect the growth of HCC1937-BRCA1 cells (Figure 1B). In addition, we also observed the synergistic effect of DNA-PK-i and PARP-i in other *BRCA1*-deficient cell lines, including UWB1.289 (UWB1) and MDA-MB-436 (Supplementary Figure S2). Collectively, these results suggest that DNA-PK-i acts together with PARP-i to suppress *BRCA1*-deficient tumor cell proliferation.

We next examined the DNA damage response. To our surprise, we did not observe DSBs generated in HCC1937 when the HR-deficient cells were treated with such low dose (1 μM) of DNA-PK-i and PARP-i (Figure 1C), nor we observed surrogate makers of DSBs, such as phospho-ATM and 53BP1 foci (Figure 1D–F), in these HCC1937 cells. These results indicate that although combination of DNA-PK-i and PARP-i treatment suppresses HCC1937 cells, this treatment does not induce DSBs in the cells.

### A fraction of DNA-PK localizes in nucleolus

**Figure 2.**
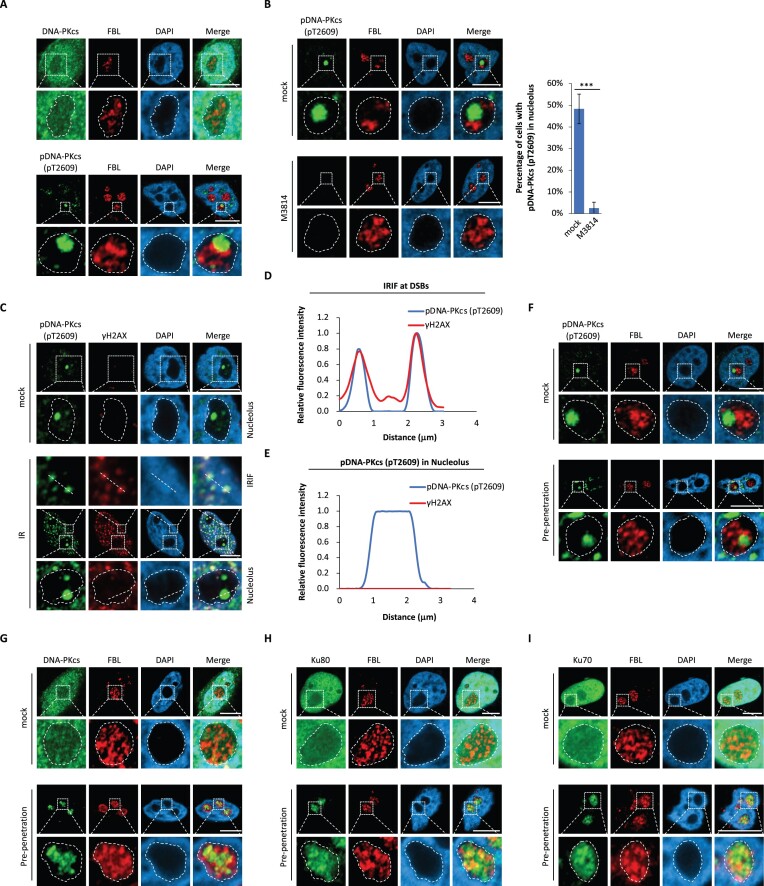
A fraction of DNA-PK localizes in nucleolus. (**A**) DNA-PKcs exists in nucleolus. DNA-PKcs and phosphorylated DNA-PKcs (pT2609) in HeLa cells were examined by IF. The dash circles indicate nucleoli. (**B**) Treatment of DNA-PK-i abolishes pDNA-PKcs (pT2609) in nucleolus. HeLa cells were pre-treated with or without M3814 (1 μM) for 24 h. pT2609 of DNA-PKcs was examined by IF. FBL was stained as the nucleolus marker. The percentage of the cells with pDNA-PKcs (pT2609) staining in nucleolus was calculated. (**C**) The phosphorylation of DNA-PKcs in nucleolus is independent of DSBs. HeLa cells were pre-treated with or without IR (20Gy). Following 24-hour recovery, pT2609 of DNA-PKcs and γH2AX were examined by IF. The localization of pT2609 of DNA-PKcs and γH2AX at DSBs on the dash line was examined in (D). IRIF, ionizing radiation-induced foci. The localization of pT2609 of DNA-PKcs and γH2AX on the dash line in nucleolus was examined in (**E**). (F–I) The activated DNA-PKcs and the DNA-PK complex localize in nucleolus. With or without detergent pre-treatment, the localization of pDNA-PKcs (pT2609) (**F**), DNA-PKcs (**G**), Ku80 (**H**) and Ku70 (**I**) in HeLa cells were examined by IF. FBL acts as the nucleolus marker. ***P < 0.001. Image bar: 10 μm.

To examine the possible mechanism by which DNA-PK-i and PARP-i induces *BRCA*-deficient cell lethality, we examined the localization of DNA-PKcs. When DNA-PKcs is activated, it is autophosphorylated at Thr2609 (40). Thus, p-Thr2609 (pT2609) is served as a surrogate marker to monitor the activated DNA-PKcs under physiological condition in cells. Interestingly, while DNA-PKcs was pan-nuclear stained by a specific anti-DNA-PKcs antibody, the activated DNA-PKcs mainly localized inside of nucleoli and formed condensates in the absence of genotoxic stress (Figure 2A). When cells were treated with M3814 or NU7441, two highly specific DNA-PK inhibitors, the activated DNA-PKcs was abolished in nucleoli (Figure 2B, Supplementary Figure S3A), suggesting that the condensation of the activated DNA-PKcs in nucleolus is dependent on the kinase activity of DNA-PKcs. Moreover, we generated the catalytically-dead mutant of DNA-PKcs (D3922A). And this mutant abolished the aggregation of the activated DNA-PKcs (Supplementary Figure S3B), further validating the requirement of the kinase activity of DNA-PKcs for its conjugation in nucleolus.

Since DNA-PK is activated in response to DSBs, the activated DNA-PK co-localized with γH2AX, a surrogate marker of DSBs as well as a substrate of DNA-PK (Figure 2C, D) (41). However, γH2AX did not exist in nucleoli, whereas activated DNA-PKcs constitutively existed in nucleoli regardless IR-induced DSBs (Figure 2C, E). Similarly, both phospho-ATM and phospho-ATR, another two markers of DSBs (42), did not localize in nucleoli either (Supplementary Figure S4), indicating that the activation of DNA-PK in nucleoli may be not induced by DSBs.

To further validate the localization of DNA-PK, we pre-penetrated cells before fixation to remove DNA-PK in nucleoplasm, and found that the activated DNA-PKcs clearly localized in nucleoli (Figure 2F, G). Using similar protocol, we found that the Ku heterodimer also localized in nucleoli (Figure 2H, I, Supplementary Figure S5). Moreover, recent studies reported that without DNA damage, Ku and DNA-PKcs, but not other cNHEJ factors, such as XRCC4 and XLF, reside in the nucleolus (30). Consistently, we did not observe the downstream effector of cNHEJ, such as Artemis in nucleolus (Supplementary Figure S6). Taken together, the DNA-PK complex may play a role in nucleolus-relevant function but not DSB repair in nucleoli.

**Figure 3.**
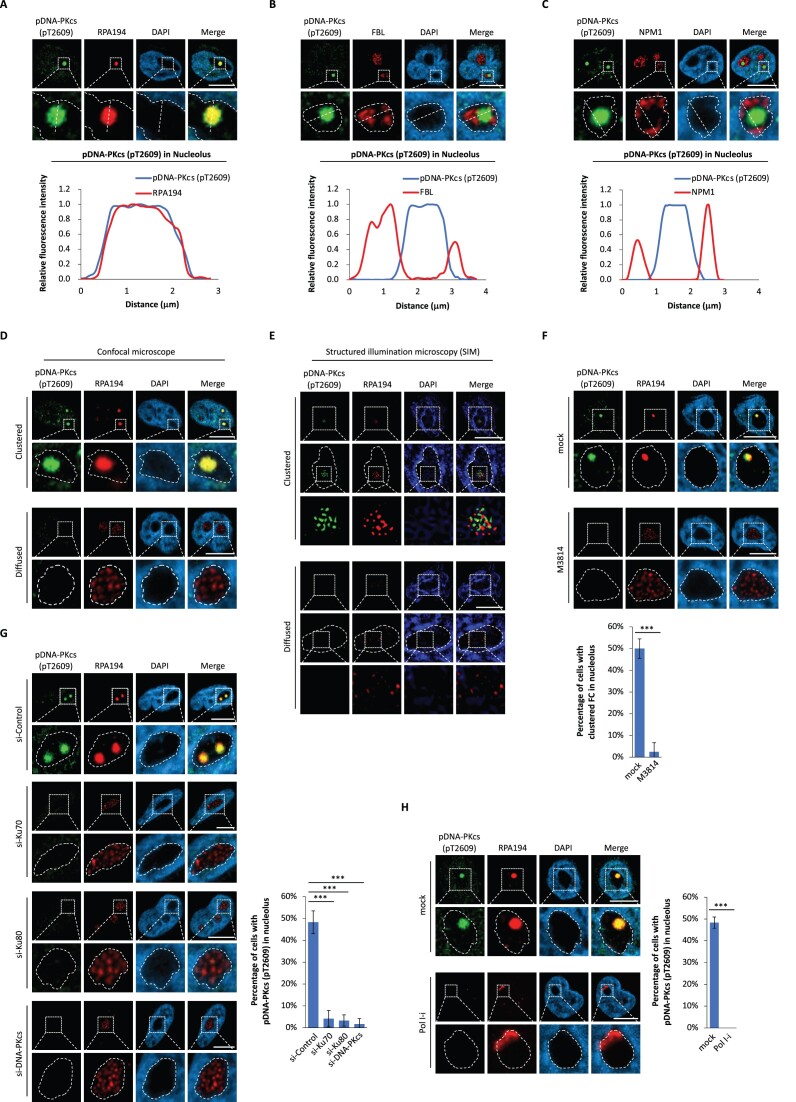
DNA-PK activation is associated with Pol I transcription in FC of nucleolus. (A–C) pDNA-PKcs (pT2609) colocalizes in FC (**A**), surrounded by DFC (**B**), and excluded from GC (**C**) in HeLa cells. RPA194 in (A), FBL in (B), NPM1 in (C) were severed as surrogate marker of FC, DFC, and GC respectively. The fluorescence intensity on the white dash line was plotted (lower panels). (D, E) The cluster of pDNA-PKcs in nucleolus was examined by a Zeiss LSM 800 Airyscan inverted confocal microscope (**D**) or a Structured illumination microscope (SIM) (**E**) in HeLa cells. (**F**) DNA-PK-i treatment abrogates the activation of DNA-PK in nucleolus, and RPA194 in FC shattered in nucleoli. HeLa cells were pre-treated with M3814 (1 μM) for 24 h. pDNA-PKcs and RPA194 in HeLa cells were examined by IF. The percentage of the cells with clustered FC in nucleolus was calculated. (**G**) Depletion of DNA-PK abrogates the conjugated form of FC in nucleolus. Following knocking-down DNA-PK subunits by siRNA in HeLa cells, pDNA-PKcs and RPA194 were examined by IF. The percentage of the cells with pDNA-PKcs staining in nucleolus was calculated. (**H**) Transient inhibition of RNA Pol I abolishes pDNA-PKcs in nucleoli. HeLa cells were treated with 1 μM BMH-21 for 2 h. pDNA-PKcs and RPA194 were examined by IF. The percentage of the cells with pDNA-PKcs staining in nucleolus was calculated. Circled area indicates the nucleolus. Image bar: 10 μm. ***P < 0.001.

It is well known that the primary function of nucleolus is to produce and assemble pre-ribosome complex. It contains three different layers with different functions, namely fibrillar center (FC) for pre-rRNA transcription, dense fibrillar component (DFC) for pre-rRNA processing, and granular component (GC) for pre-ribosome assembly (Supplementary Figure S7). To explore the functions of DNA-PK in nucleolus, we examined the detailed localization of DNA-PK in nucleolus. With RPA194, FBL and NPM1 as biomarkers for FC, DFC, and GC respectively, we examined and found that the activated DNA-PK was mainly localized in FC (Figure 3A–C, Supplementary Figure S8).

Intriguingly, with immunofluorescence staining of RPA194, we observed two types of FC patterns in nucleolus: (a) the clustered FC pattern, in which RPA194 forms a large cluster that colocalized with the activated DNA-PKcs in nucleolus (Figure 3D); (b) the relatively diffused FC pattern, in which no activated DNA-PKcs was observed in nucleolus (Figure 3D). This observation is consistent with earlier studies that the different FC patterns have been observed (43). They observed the preference of the clustered FC pattern in nucleolus in G1 phase, suggesting that the clustered FC pattern may facilitate rDNA transcription (43). The FC itself is a unique type of nuclear condensate with rDNA and transcription machinery. It has been shown that the rDNA units on each chromosome range from 1–3 to >140 (44). It is possible that some nucleolus organizer regions (NORs) may contain more actively transcribed rDNA units. Moreover, we used SIM to observe the details of the clustered FC and found that it is composed of numerous small foci of RNA transcription machinery (Figure 3E). It is possible that the FCs in NORs may encounter each other, and fuse into a large center, where pDNA-PKcs may facilitate pre-rRNA transcription and processing.

Since FC is the center for Pol I-dependent pre-rRNA transcription, we ask if DNA-PK activation is associated with Pol I-dependent pre-rRNA transcription. DNA-PK-i treatment or knockdown each subunit of the DNA-PK complex by siRNA abrogated the activation of DNA-PK in nucleoli in HeLa cells. Moreover, RPA194 in FC shattered in nucleoli as well (Figure 3F, G). These results were validated in two additional cell lines, UWB1 and MDA-MB-436 (Supplementary Figure S9). In contrast, suppression of Pol I-mediated rDNA transcription by Pol I-i (BMH-21) treatment induced ribosomal biogenesis stress that RPA194, the catalytic core subunit of Pol I complex, relocated to the cap regions of nucleoli (45). Interestingly, nucleolar localization of activated DNA-PKcs was abolished by Pol I-i treatment in HeLa cells (Figure 3H), which was further validated in two other cell lines, UWB1 and MDA-MB-436 (Supplementary Figure S10). These results suggest that active transcription of pre-rRNA may associate with the nucleolar localization of DNA-PK.

We further asked whether DNA-PKcs localization in nucleolus is dependent on Ku proteins or Pol I transcription. We observed that loss of Ku subunits largely reduced the localization of DNA-PKcs in the nucleolus (Supplementary Figure S11), suggesting that the Ku subunits are required for targeting DNA-PKcs to the proper locations in the nucleolus. In addition, we found that DNA-PK did not interact with RPA194 (Supplementary Figure S12A), and suppression of pre-rRNA biogenesis by Pol I-i (BMH-21) treatment abolished the nucleolar localization of DNA-PK (Supplementary Figure S12B–D), suggesting that the localization of DNA-PK in nucleolus is dependent on pre-rRNA biogenesis. Moreover, we found that knockdown the catalytic core component (RPA194) of Pol I complex by siRNA abrogated the nucleolar localization of DNA-PK (Supplementary Figure S12E–G), suggesting that Pol I-mediated rDNA transcription is required for the localization of DNA-PK in nucleolus.

Next, we examined whether DNA-PK binds and is activated by ribosome DNA (rDNA). However, using CHIP-qPCR assays, we found that DNA-PKcs did not bind rDNA. RPA194, the catalytic subunit of RNA Polymerase I complex (Pol I) which synthesizes pre-rRNA via rDNA transcription, was used as a positive control (Supplementary Figure S13). These results indicate that the activation of DNA-PK in nucleolus may be not induced by rDNA.

### DNA-PK recognizes pre-rRNA for rRNA biogenesis

**Figure 4.**
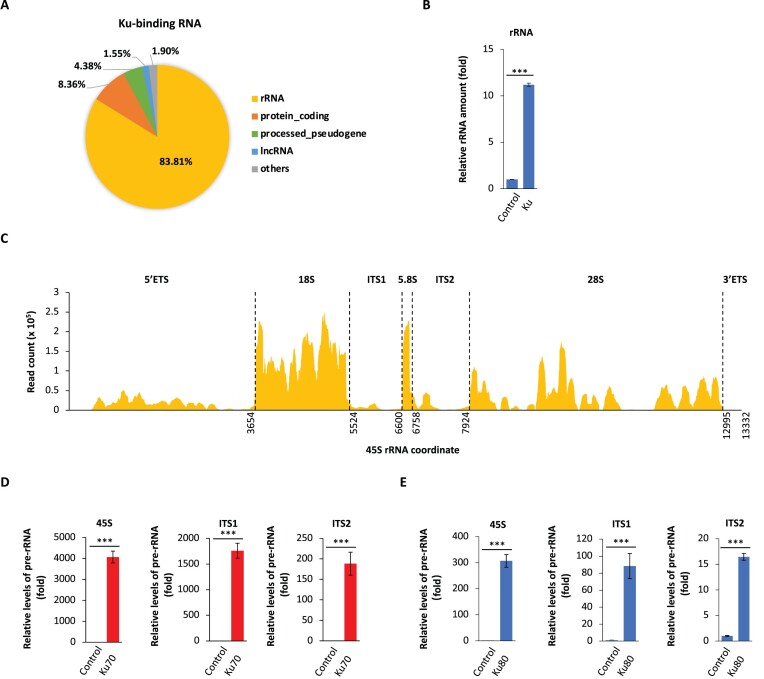
The Ku complex recognizes pre-rRNA. (**A**) The Ku complex-associated RNA was examined by PAR-CLIP assays. SFB-Ku80 was expressed in HEK293T cells, which were used for the assays. The RNA species isolated from the cells are shown. (**B**) The Ku complex associates with rRNA. The relative fold of rRNA enrichment is calculated by the percentage of rRNA reads in the RNA sequencing times the absolute amount of total RNA purified in the PAR-CLIP assays. The rRNA enriched from the Ku80 purification is compared to that from HEK293T cells expressing SFB-vector. (**C**) The RNA sequencing reads from the purification of the Ku complex in HEK293T cells were mapped onto 45S pre-rRNA. (D, E) Ku70 (**D**) and Ku80 (**E**) associate with pre-rRNA. Endogenous Ku70 or Ku80 from HEK293T cells was immunoprecipitated, q-PCR was performed to examine the enrichment of pre-rRNA. Irrelevant IgG was used as the negative control. ***P < 0.001.

Since the Ku heterodimer is also required for the activation of DNA-PKcs in nucleolus (Figure 3G), we performed photoactivatable-ribonucleoside-enhanced crosslinking and immunoprecipitation (PAR-CLIP) assay to search for the functional partner of the Ku70/80 complex in nucleolus. Interestingly, we found that majority RNA species-associated with Ku70/80 is pre-rRNA (Figure 4A–C, Supplementary Table S1). To validate the results from PAR-CLIP assays, we particularly designed primers targeting the 5′ETS, ITS1 and ITS2 regions of pre-rRNA, and performed RT-qPCR. Again, we found that the Ku complex clearly recognized pre-rRNA (Figure 4D, E). Since rRNA is very abundant in the cell, we have examined other relatively abundant non-coding RNAs, such as 7SK and MALAT1, and did not find that the Ku complex interacts with these RNA species (Supplementary Figure S14A). In addition, we included PARP10, an RNA-binding protein with an RNA recognition motif (46), to further examine the binding specificity of Ku complex associated pre-rRNA. We observed that the Ku complex clearly recognized pre-rRNA (Figure 4D, E), but PARP10 did not interact with pre-rRNA (Supplementary Figure S14B). Thus, these results suggest that the Ku complex specifically associates with pre-rRNA.

Ku70 is also known as a DNA binding protein, and its C-terminus (amino acids 439–609) mediates the DNA binding (47). Thus, we generated the truncated Ku70-1-438 mutant that lacks DNA-binding ability. We found that only the full length Ku70 but not the truncation mutant was able to recognize pre-rRNA (Supplementary Figure S15). These results suggest that the Ku proteins may use the same nucleic acid-binding motif to interact with either DNA or pre-rRNA. It is likely that pre-rRNA recognized by the Ku complex may mediate the activation of DNA-PKcs in nucleolus. In addition, we performed RIP assay coupled with RT-qPCR assays, and found that similar to the Ku proteins, DNA-PKcs associated with pre-rRNA (Supplementary Figure S16).

**Figure 5.**
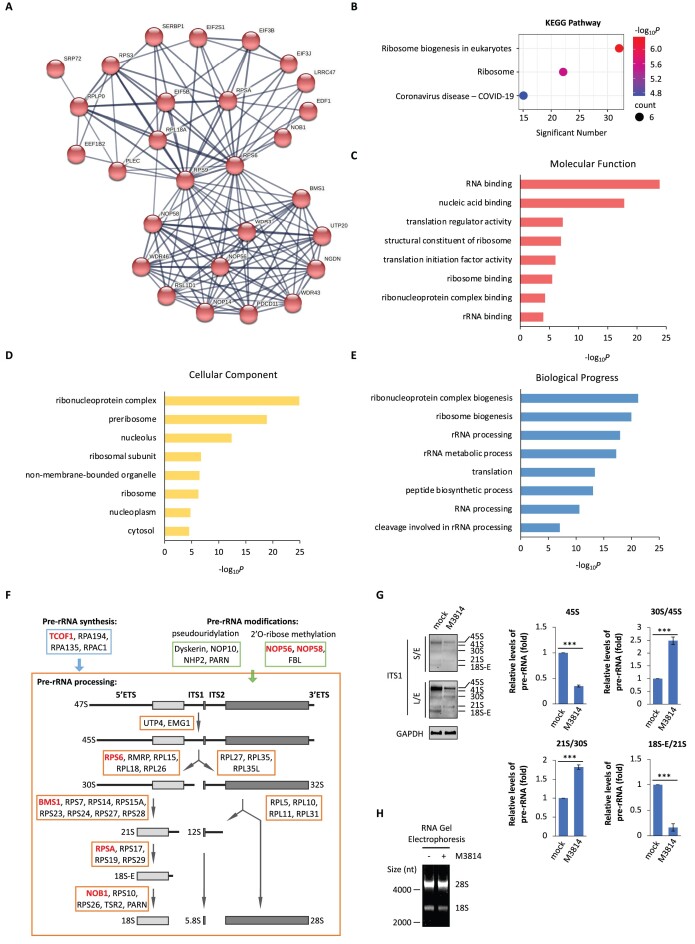
DNA-PK regulates multiple steps of ribosome biogenesis in nucleolus. (**A**) Network presentation of physical connections between proteins in the largest cluster from the substrates of DNA-PK in HeLa cells. (**B**) The KEGG pathway analysis of the substrates of DNA-PK in HeLa cells. (C–E) The substrates of DNA-PK are classified by molecular function (**C**), cellular components (**D**), and biological progress (**E**). (**F**) The ribosomal proteins identified (marked in red) from DNA-PK phosphoproteomics in HeLa cells are mapped to rRNA biogenesis pathway, including pre-rRNA synthesis, pre-rRNA modifications, and pre-rRNA processing. (**G**) DNA-PK-i treatment impairs pre-rRNA biogenesis. HeLa cells were treated with M3814 (1 μM) for 24 h, Northern blotting was performed to examine pre-rRNA with probes targeting ITS1 region (left panel). GAPDH was used as loading control. The relative levels of 45S, 30S/45S, 21S/30S and 18S-E/21S pre-rRNA were measured (right panels). S/E, short exposure; L/E, long exposure. (**H**) DNA-PK-i treatment alone partially reduces the production of both 18S and 28S rRNAs. Total RNA was isolated from HeLa cells treated with or without M3814 (1 μM) for 24 h. The RNA was visualized by RNA gel electrophoresis. ***P < 0.001.

Since DNA-PKcs is a PI-3 like protein kinase, we searched for the substrates of DNA-PKcs in nucleoli. We performed phosphoproteomics with cells treated with or without DNA-PK inhibitor, and identified DNA-PK phosphorylation sites on 924 DNA-PKcs substrates (Supplementary Tables S2 and S3). To further explore the potential function of DNA-PK, we analyzed the physical connections between the identified proteins from DNA-PK phosphoproteomics (Supplementary Figure S17), and we found that 28 ribosomal proteins formed the largest network with functions involved in rRNA biogenesis (Figure 5A). With the KEGG pathway analysis, DNA-PK substrates, under physiological condition, are mainly involved in ribosome biogenesis but not DSB repair (Figure 5B). In addition, we performed GO analysis and obtained similar results (Figure 5C - E). Additionally, we mapped the DNA-PK phosphorylated proteins into rRNA biogenesis pathways and observed that 7 of them were previously validated ribosomal proteins or ribosome-associated proteins participated in most steps of rRNA biogenesis, including: (a) TCOF1 in pre-rRNA synthesis; (b) NOP56 and NOP58 in pre-rRNA modification; (c) RPS6, BMS1, RPSA and NOB1 in pre-rRNA processing (Figure 5F). Taken together, these results suggest the potential role of DNA-PK in regulating rRNA biogenesis, including pre-rRNA synthesis, modification, and processing.

rRNA is transcribed from rDNA loci as 47S pre-rRNA by RNA Pol I. Subsequently, 47S pre-rRNA is processed to 45S, which is further digested into 30S and 32S intermediates. 30S is cleavage into 21S that is converted into 18S-E, the precursors of 18S rRNA. Meanwhile, 32S pre-rRNA encompasses several cleavage reactions and is digested into 5.8S and 28S rRNA (48) (Figure 5F). To study the biological function of DNA-PK in nucleolus, we performed northern blots with various probes covering different regions of 47S/45S pre-rRNA. With the treatment of DNA-PK-i, we observed a clear reduction of 45S pre-rRNA (Figure 5G). Moreover, among the precursors of 18S rRNAs, both 30S and 21S pre-rRNA were accumulated, but the levels of 18S-E pre-rRNA was sharply reduced (Figure 5G). In addition, the levels of 18S and 28S rRNAs were partially reduced when the cells were treated with DNA-PK-i (Figure 5H). Thus, these results suggest loss of activated DNA-PK suppresses not only the 47S pre-rRNA transcription but also the processing of precursors of 18S rRNAs.

To further elucidate the biological functions of the phosphorylation events in nucleolus mediated by DNA-PK, we examined RPS6, a substrate of DNA-PK identified by mass spectrometry (Supplementary Tables S2 and S3). With the treatment of DNA-PK-i, we validated that DNA-PK mediated the phosphorylation of S235/S236 on RPS6 (Supplementary Figure S18A). Moreover, knockdown each subunit of the DNA-PK complex by siRNA abrogated RPS6 phosphorylation on S235/S236 (Supplementary Figure S18B). Interestingly, a recent report demonstrates that RPS6 S235/S236 phosphorylation is required for 30S pre-rRNA processing (49). Consistently, we observed the drastic accumulation of 30S pre-rRNA upon DNA-PK inhibitor treatment (Figure 5G). Thus, our results suggest that the regulation of 30S pre-rRNA processing may be at least in part via DNA-PK-mediated phosphorylation of RPS6 at S235 and S236, which further validates that DNA-PK regulates rRNA biogenesis by phosphorylating ribosomal proteins.

### DNA-PKcs-i acts synthetically with PARP-i to suppress rRNA biogenesis and translation

**Figure 6.**
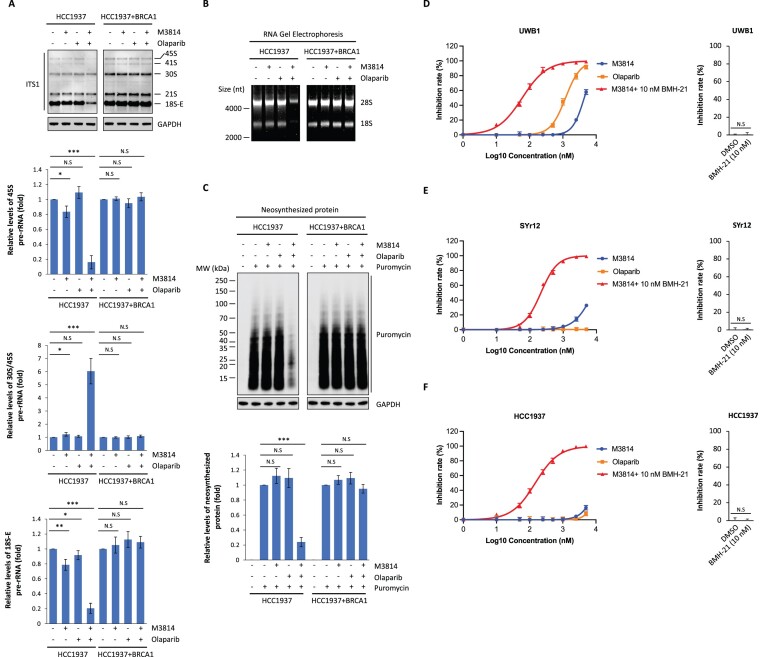
DNA-PKcs-i acts synthetically with PARP-i to suppress ribosome biogenesis and protein translation. (**A**) M3814 acts together with olaparib to suppress rRNA biogenesis. Northern blot analysis of pre-rRNA synthesis and processing on HCC1937 or HCC1937-BRCA1 cells with the pre-treatment of M3814 (1 μM) and/or olaparib (1 μM) for 24 h (upper panel). Probes targeting ITS1 region were used. GAPDH was used as loading control. The relative levels of pre-rRNA were measured (lower panels). (**B**) M3814 acts together with olaparib to reduce the production of both 18S and 28S rRNAs. Total RNA was isolated from HCC1937 or HCC1937-BRCA1 cells with the pre-treatment of M3814 (1 μM) and/or olaparib (1 μM) for 24 h. The RNA was visualized by RNA gel electrophoresis. (**C**) Protein synthesis is suppressed by the treatment of M3814 and olaparib. HCC1937 or HCC1937-BRCA1 cells were pre-treated with M3814 (1 μM) and/or olaparib (1 μM) for 24 h. The relative neosynthesized protein levels were measured (lower panel). (**D**) DNA-PK-i (M3814) and Pol I-i (BMH-21) act synergistically to suppress the growth of UWB1 cells. UWB1 cells were treated with the indicated dose of M3814, olaparib, or M3814 together with 10 nM of BMH-21 for 7 days. Cell growth was measured using CellTiter-Glo assays. The single arm of BMH-21 (10 nM) treatment in the cell viability assays is shown in the right panel. Average cell growth suppression is presented as mean ± SD. (E, F) Combination treatment of DNA-PK-i (M3814) and Pol I-i (BMH-21) suppresses the growth of *BRCA*-deficient PARP-i resistant tumor cells. SYr12 (**E**) or HCC1937 (**F**) cells were treated with the indicated dose of M3814, olaparib, or M3814 together with 10 nM of BMH-21 for 7 days. Cell growth was measured using CellTiter-Glo assays. The single arm of BMH-21 (10 nM) treatment in the cell viability assays is shown in the right panel. Average cell growth suppression is presented as mean ± SD. *P < 0.1, **P < 0.01, ***P < 0.001.

In addition to DNA-PK, recent studies show that PARP1 also plays a key role for ribosome biogenesis (50,51). PARP-i treatment clearly suppresses pre-ribosome complex assembly in nucleolus (51). Since we found that DNA-PK-i and PARP-i synergistically inhibited *BRCA*-deficient tumor cells growth without generating obvious DSBs, we ask if this cellular phenomenon is caused by abrogation of ribosome biogenesis. With Northern blots, we observed that the levels of rRNA biogenesis, including both pre-rRNA synthesis and pre-rRNA processing, were significantly decreased upon DNA-PK-i (M3814) and PARP-i (olaparib) co-treatment in HCC1937 cells (Figure 6A). Using the different DNA-PK-i, NU7441, we confirmed these results (Supplementary Figure S19A). Since the transcription of rRNA is quite different among different cancer cells, the level of 45S pre-rRNA was relatively low in HCC1937 cells. Nevertheless, the reduction of 45S pre-rRNA and 18S-E pre-rRNA suggests that both pre-rRNA transcription and processing are impaired when HCC1937 cells are treated with DNA-PK-i and PARP-i. Furthermore, because reductions in rRNA biogenesis should presumably affect ribosome biogenesis and its-dependent protein translation function, we determined the effects of DNA-PK-i and PARP-i on the levels of 18S and 28S mature rRNAs using RNA Gel Electrophoresis. We also examined newly synthesized proteins using puromycin incorporation assays (35). We observed that both ribosome biogenesis and protein translation were sharply decreased upon DNA-PK-i (M3814) and PARP-i (olaparib) co-treatment especially in HCC1937 cells (Figure 6B, C). With another DNA-PK-i, NU7441, we validated these results (Supplementary Figure S19B). Taken together, these results suggest that DNA-PK-i acts synthetically with PARP-i to suppress rRNA biogenesis, protein translation, and *BRCA*-deficient tumor cell proliferation.

To further explore the potential function of DNA-PK-i in cancer treatment, we treated *BRCA*1-deficient UWB1 ovarian cancer cells with combination of low dose DNA-PK-i (M3814) with Pol I-i (BMH-21). Low dose of M3814 treatment alone did not suppress UWB1 cell growth. However, combination treatment of M3814 and BMH-21 additively suppressed UWB1 cells growth (Figure 6D, Supplementary Figure S20A). And this combination treatment synergistically suppressed the cell growth of UWB1 with better efficacy than PARP-i (olaparib) treatment (Figure 6D).

Although several PARP inhibitors have been approved for the treatment of breast and/or ovarian cancers with *BRCA* mutations, like other targeted therapies, those patients who showed initial response to PARP-i often develop resistance, and relapsed disease is commonly observed. UWB1.289-SYr12 (SYr12) cells were derived from long-term culturing of the parental UWB1 cells in the presence of olaparib (39), thus was resistant to olaparib treatment (Figure 6E). Interestingly, SYr12 cells were still hypersensitive to the combination treatment of M3814 and BMH-21 with various doses (Figure 6E, Supplementary Figure S20B). Furthermore, we observed the synergistic effect of DNA-PK-i and Pol-i treatment on suppressing PARP-i resistant HCC1937 cells growth (Figure 6F). Thus, these results indicate that targeting rRNA biogenesis can be an alternative strategy to overcome PARP-i resistance during *BRCA*-deficient cancer treatment.

## Discussion

In this study, we have shown that DNA-PK plays an important role in pre-rRNA biogenesis. With Northern blotting assays, we found that DNA-PK-i treatment not only suppressed the transcription of 45S pre-rRNA, but also abolished pre-rRNA processing steps, particularly for 18S rRNA precursors. We observed the up-regulation of 30S and 21S pre-rRNA but down-regulation of 18S-E pre-rRNA, suggesting that processing of 30S and 21S is partially disrupted. Of note, different cancer cells have different pre-rRNA transcription and processing patterns. However, we always observe the reduction of 45S and 18S-E pre-rRNA, suggest that DNA-PK regulates both transcription and processing of pre-rRNA. Consistently, we found that a number of pre-rRNA transcription and processing factors were substrates of DNA-PK. It is likely that phosphorylation of these factors is involved in pre-rRNA transcription and processing.

Pre-rRNA was transcribed by Pol I at FC of nucleolus. Consistently, we found that the activated DNA-PK localized at FC. Since we did not observe obvious DSBs in nucleolus, it is unlikely that the Ku heterodimer recognizes DNA ends for the activation of DNA-PKcs in nucleolus. Instead, we have shown that the Ku proteins were able to recognize pre-rRNA. Moreover, the transcription of pre-rRNA per se controls the localization of activated DNA-PK in nucleolus (Figure 3). Thus, it is possible that this molecular event triggers the activation of DNA-PKcs, which provides positive feedback to promote pre-rRNA synthesis. Future studies *in vitro* binding assays are needed to examine if the interactions between DNA-PKcs and pre-rRNA is mediated the Ku complex. Moreover, *in vitro* kinase assays will demonstrate if pre-rRNA is able to activate DNA-PKcs. With current results, we postulate that the interaction between the Ku70/80 heterodimer and pre-rRNA could be the key factor to activate the kinase activity of DNA-PKcs.

In addition to pre-rRNA transcription, DNA-PK regulates pre-rRNA processing. Interestingly, we found that the activated DNA-PK mainly localized in FC but not in DFC or GC. However, unbiased mass spectrometry analyses show that a number of processing factors are phosphorylated by DNA-PK, and majorities of these processing factors localize in DFC and GC. It is possible that DNA-PK phosphorates these factors via ping-pong reactions, and the phosphorylated processing factors mediate pre-rRNA processing in DFC and GC.

Consistent with our findings, a recent study also shows that DNA-PK regulates rRNA biogenesis (30). Slightly different from our studies, they show that DNA-PK has U3 snoRNA dependent functions during ribosome biogenesis. However, in the PAR-CLIP assays, we found that Ku proteins mainly associate with pre-rRNA. Our study provides the first evidence that Ku proteins recognize pre-rRNA that may facilitate the activation of DNA-PKcs for pre-rRNA biogenesis. Thus, it is possible that DNA-PK may regulate pre-rRNA biogenesis at multiple steps.

In this study, we also found that DNA-PK-i and PARP-i synergistically suppressed *BRCA*-deficient tumor cell growth. In particular, we have applied very low dose of DNA-PK-i and PARP-i, and observed synergistical effects on tumor cell growth suppression. Although both DNA-PK and PARP1 participate in DSB repair, we did not observe obvious DSBs in such low dose treatment. Instead, we found that ribosome biogenesis was abrogated. A recent study shown that activated PARP1 ADP-ribosylates DDX21, an RNA helicase that localizes to nucleoli and promotes rDNA transcription when ADP-ribosylated, and treatment with PARP-i reduces DDX21 nucleolar localization, rDNA transcription and ribosome biogenesis (51). Genetically knockout PARP1 in drosophila also suppresses ribosome biogenesis (50), which strongly suggests PARP1 acting as a key regulator for ribosome biogenesis. Similar to PARP-i treatment, DNA-PK-i treatment also impairs ribosome biogenesis, albeit at different pre-rRNA steps. Thus, combination of DNA-PK-i and PARP-i treatment synergistically impairs ribosome biogenesis, which leads to tumor cell apoptosis. Consistent with this line of evidence, we also combined Pol I-i and DNA-PK-i, which also induces *BRCA*-deficient tumor cell growth suppression. In fact, Pol I-i are currently in clinical trials for *BRCA*-deficient tumor treatment, and our results show that DNA-PK-i is able to sensitize *BRCA*-deficient tumor cells to Pol I-i treatment, in particular for PARP-i resistant tumor cells. However, regarding other PARP-i-resistant and *BRCA*-deficient cells, it is very rare to find. There are only a few PARP-i-resistant and *BRCA*-deficient cell lines for researchers to study. Most PARP-i-resistant and *BRCA*-deficient tumors are from clinically treated patients, and it is currently beyond our capability to obtain such clinical resources. Nevertheless, we have demonstrated two important PARP-i-resistant and *BRCA*-deficient cell lines in current analyses (Figure 6E, F). Future clinical trials would validate our working models.

A previous report found that PARP1 and DNA-PK are involved in the DNA damage-induced rRNA synthesis interruption, which focuses on the roles of PARP1 and DNA-PK in coordinating both DNA damage response and rRNA synthesis under genotoxic stress (52). However, in our study, we found that simultaneous treatment of low dose of DNA-PK-i and PARP-i induces synthetic lethality in a triple-negative breast cancer cell line lacking BRCA1, which is elicited through defects in pre-rRNA biogenesis, but not DNA damage response. Therefore, our current study mainly focuses on the novel roles of PARP1 and DNA-PK in rRNA biogenesis, in the absence of genotoxic stress.

In this study, we have examined the synergistic effect of DNA-PK with PARP1 in a panel of *BRCA1*-deficient and proficient cell lines, including HCC1937/HCC1937-BRCA1, UWB1/UWB1-BRCA1 and MDA-MB-436 (*BRCA1*-deficient) /MDA-MB-231 (*BRCA1*-proficient) cells. We observed that *BRCA1*-deficient but not *BRCA1*-proficient cells are hypersensitive to DNA-PK-i and PARP-i treatment (Figure 1B, Supplementary Figure S2). These results suggest that DNA-PK-i acts synthetically with PARP-i to suppress *BRCA1*-deficient tumor cell proliferation. In addition, it has been shown that BRCA1 also regulates Pol I-mediated transcription (53). Although we did not observe obvious pre-rRNA biogenesis defects in *BRCA1*-deficient cells, these *BRCA1*-deficient cells often grow very slowly, indicating that minor defects on ribosome biogenesis may exist. Consistently, previous studies show that both BRCA1 and PARP1 regulate pre-rRNA biogenesis (50,51,53). Here, in our study, we identified that DNA-PK regulates pre-rRNA synthesis via the phosphorylation of a number of substrates. Thus, BRCA1, PARP1 and DNA-PK may act together in pre-rRNA biogenesis. Loss of only one or two factors, ribosomal biogenesis may be partially affected, and tumor cells may still be viable. However, depletion of all three factors leads to severe suppression of ribosomal biogenesis. Thus, *BRCA1*-deficient tumor cells are hypersensitive to the combination of DNA-PK-i and PARP-i treatment.

Moreover, when exposed to low dose of DNA-PK-i and PARP-i treatment, we found that ribosome biogenesis was impaired in *BRCA1*-deficient but not *BRCA1*-proficient cells. Again, we did not identify any obvious DSBs in these tumor cells, suggesting that under the low dose treatment of DNA-PK-i and PARP-i, it is ribosome biogenesis impairment but not induced DSBs suppress *BRCA1*-deficient tumor cell growth. The ribosome biogenesis defects are associated with the reduction of protein translation. Thus, it is very likely that lacking newly synthesized proteins triggers *BRCA*-deficient cancer cell apoptosis when these cells are treated with DNA-PK-i and PARP-i. It is also possible that ribosome biogenesis and processing may sever as biomarkers to guild the cancer clinical trials of DNA-PK-i in future.

## Supplementary Material

gkae316_Supplemental_Files

## Data Availability

All datasets have been deposited in the GEO Datasets under the GEO accession number GSE219220 and ProteomeXchange Consortium via the iProX repository with the dataset identifier PXD039454.
