# Supplementary material for: DNA-PK participates in pre-rRNA biogenesis independent of DNA double-strand break repair
Source: Nucleic Acids Res. 2024 Apr 29;52(11):6360–75. doi: 10.1093/nar/gkae316 (PMC11194077; doi:10.1093/nar/gkae316)
Supplement: gkae316_Supplemental_Files [file gkae316_supplemental_files.zip › Supplementary data.pdf]

Supplementary Information for

DNA-PK participates in pre-rRNA biogenesis independent of DNA double-strand break repair

Peng Li[1,2,3], Xiaochen Gai[1,2,3], Qilin Li[1,2,3], Qianqian Yang[1,2,3] and Xiaochun Yu[1,2,3]*

[1]Westlake Laboratory of Life Sciences and Biomedicine, Hangzhou, Zhejiang, China.

[2]School of Life Sciences, Westlake University, Hangzhou, Zhejiang, China.

[3]Institute of Basic Medical Sciences, Westlake Institute for Advanced Study, Hangzhou, Zhejiang, China.

*To whom correspondence should be addressed. Email: yuxiaochun@westlake.edu.cn

This file includes:

Supplementary Figures. S1 to S20

Other Supporting Online Material for this manuscript includes the following:

Supplementary Table S1. The full PAR-CLIP analysis results.
Supplementary Table S2. List of DNA-PK phosphorylation sites on substrates.
Supplementary Table S3. List of DNA-PK phosphoproteins.

Supplementary Figure S1

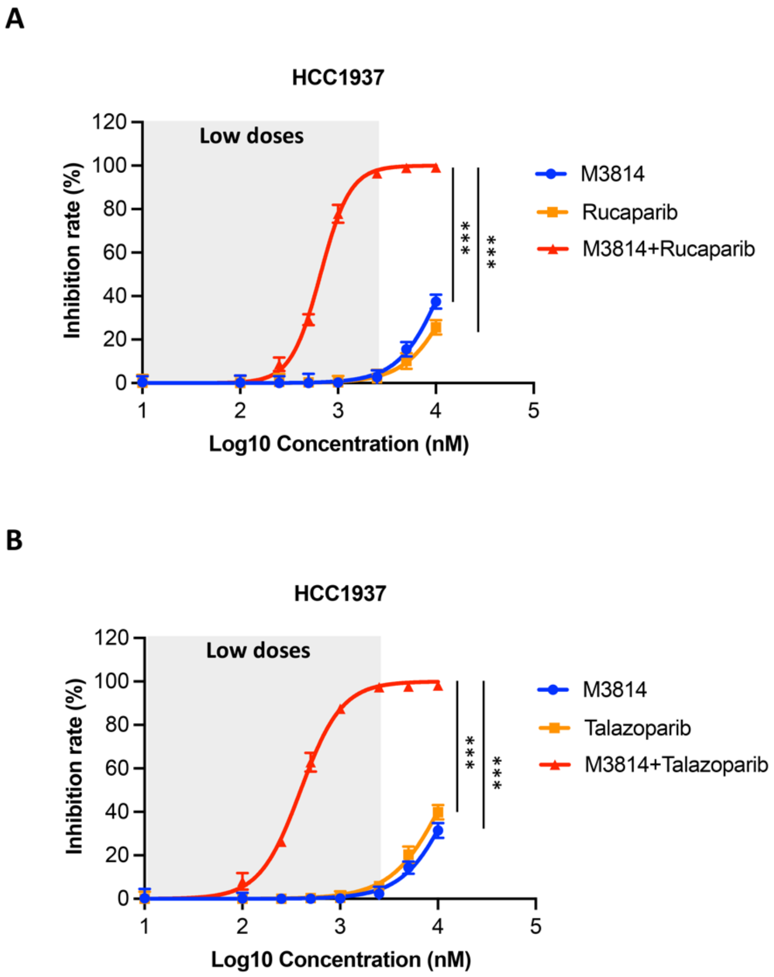

Supplementary Figure S1. DNA-PK-i acts synthetically with PARP-i to suppress the growth of HCC1937 cells.

HCC1937 cells were treated with the indicated dose of M3814 and/or PARP-i [rucaparib (**A**) or talazoparib (**B**)] for 7 days. Cell growth was measured with CellTiter-Glo assays. Average cell viability is presented as mean ± SD. ***$p < 0.001$.

Supplementary Figure S2

A

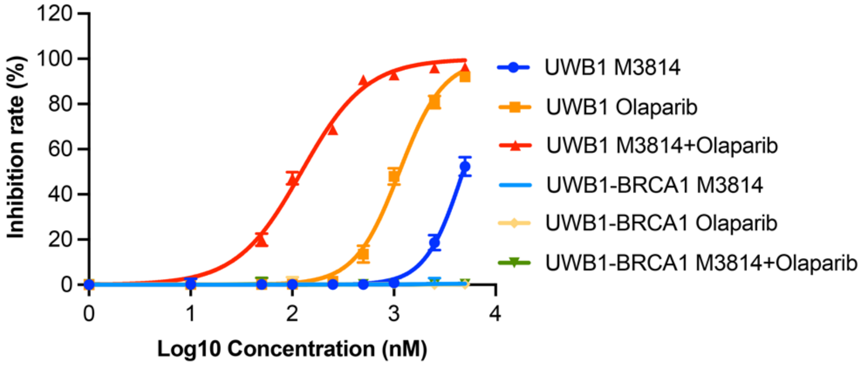

B

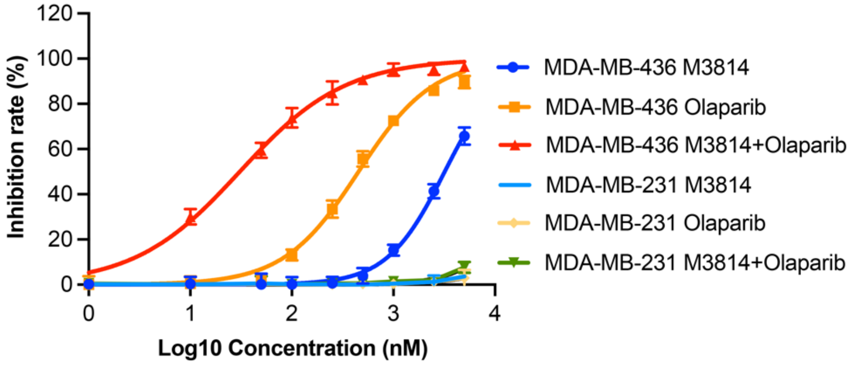

Supplementary Figure S2. DNA-PK-i acts synthetically with PARP-i to suppress *BRCA1*-deficient tumor cell proliferation.

(**A**) UWB1 or UWB1-BRCA1 cells were treated with the indicated dose of M3814 and/or olaparib for 7 days. Cell growth was measured with CellTiter-Glo assays. Average cell viability is presented as mean ± SD. (**B**) MDA-MB-436 or MDA-MB-231 cells were treated with the indicated dose of M3814 and/or olaparib for 7 days. Cell growth was measured with CellTiter-Glo assays. Average cell viability is presented as mean ± SD.

Supplementary Figure S3

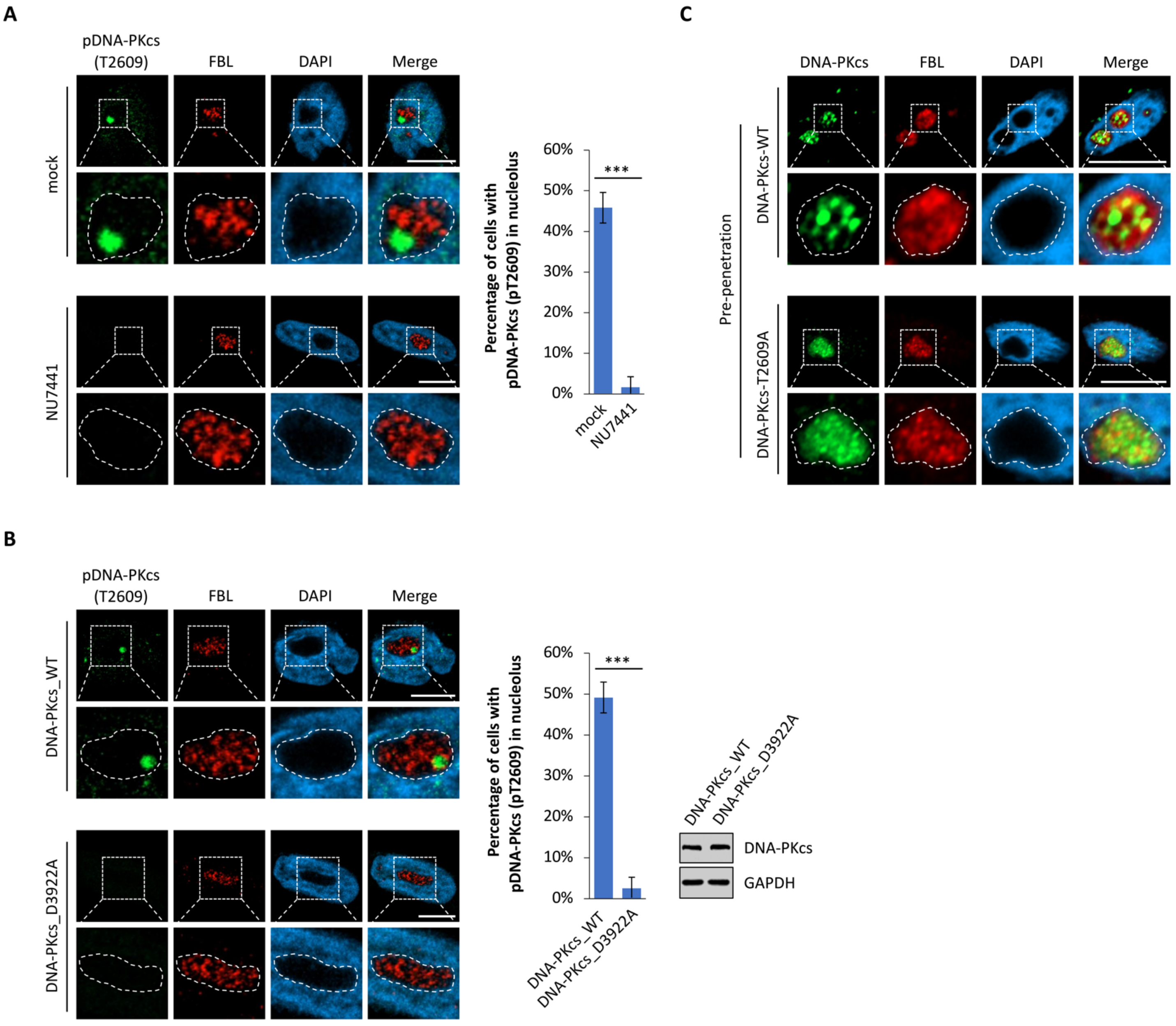

Supplementary Figure S3. DNA-PK-i or DNA-PKcs mutants abolish pDNA-PKcs (pT2609) in nucleolus.

(**A**) Treatment of DNA-PK-i abolishes pDNA-PKcs (pT2609) in nucleolus. HeLa cells were pre-treated with or without NU7441 (1 μM) for 24 hours. pT2609 of DNA-PKcs was examined by IF. FBL acts as the nucleolus marker. Circled area indicates the nucleolus. The percentage of the cells with pDNA-PKcs (pT2609) staining in nucleolus was calculated. (**B**) The kinase-dead DNA-PKcs mutant (D3922A) abolishes the phosphorylation of DNA-PKcs at T2609. DNA-PKcs-KO HeLa cells were reconstituted with DNA-PKcs-WT or DNA-PKcs-

D3922A mutant. pT2609 of DNA-PKcs was examined by IF. FBL acts as the nucleolus marker. Circled area indicates the nucleolus. The percentage of the cells with pDNA-PKcs (pT2609) staining in nucleolus was calculated. DNA-PKcs expression in the two cell lines was examined by Western blot. (**C**) DNA-PKcs-T2609A mutant reduces the DNA-PKcs aggregation in nucleolus. DNA-PKcs-KO HeLa cells were reconstituted with DNA-PKcs-WT or DNA-PKcs-T2609A mutant. With detergent pre-treatment, DNA-PKcs was examined by IF. FBL acts as the nucleolus marker. Circled area indicates the nucleolus. Image bar: 10 μm. ***$p < 0.001$.

Supplementary Figure S4

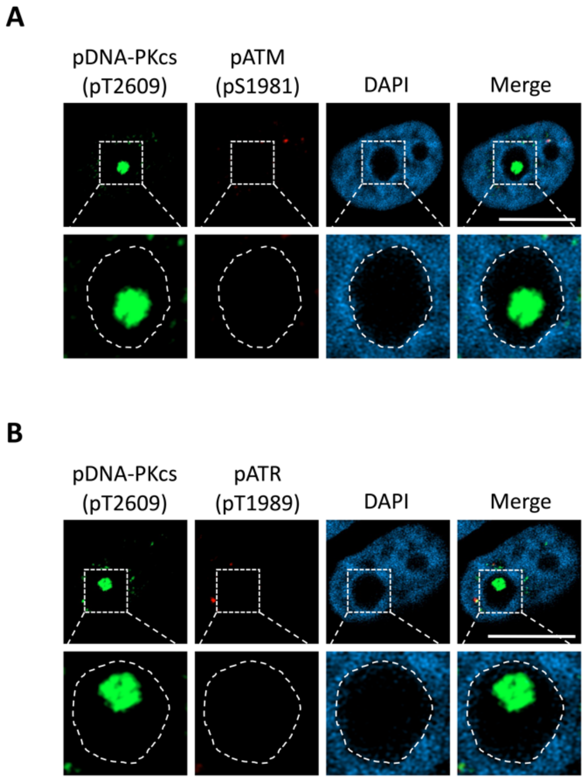

Supplementary Figure S4. The phosphorylation of DNA-PKcs in nucleolus is independent of DSBs.

(**A**) The phosphorylation of DNA-PKcs does not colocalize with pATM (pS1981) in nucleolus. pT2609 of DNA-PKcs and pS1981 of ATM were examined by IF. Circled area indicates the nucleolus. (**B**) The phosphorylation of DNA-PKcs does not colocalize with pATR (pT1989) in nucleolus. pT2609 of DNA-PKcs and pT1989 of ATR were examined by IF. Circled area indicates the nucleolus. Image bar: 10 μm.

Supplementary Figure S5

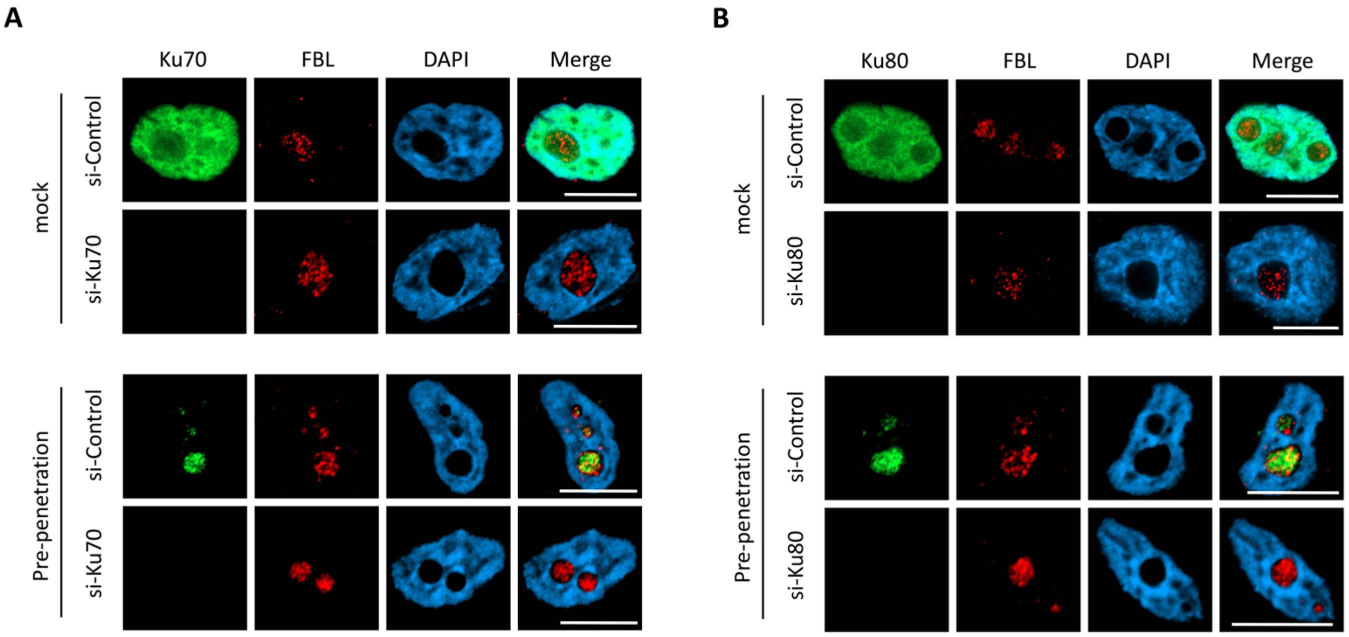

Supplementary Figure S5. Specific Ku70/80 stainings in HeLa cells.

(**A, B**) Following knocking-down Ku proteins by siRNA in HeLa cells, the cells were pre-treated with or without detergent. Ku70 (**A**) or Ku80 (**B**) was examined by IF. FBL acts as the nucleolus marker. Image bar: 10 μm.

Supplementary Figure S6

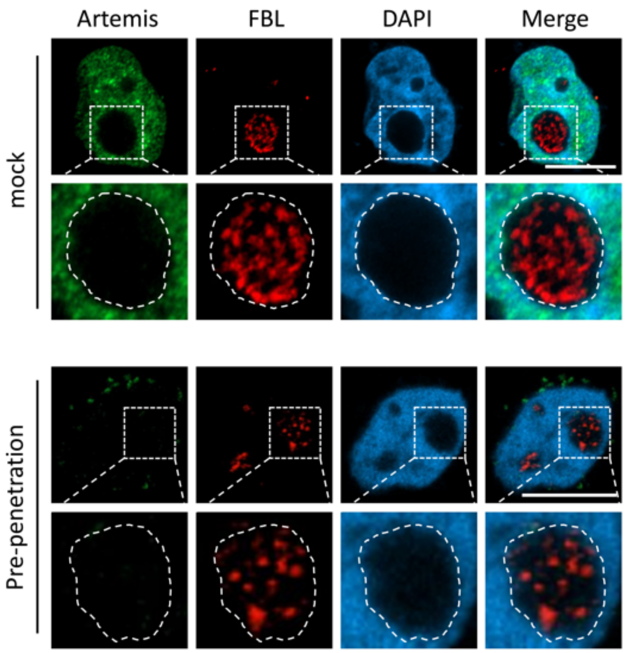

Supplementary Figure S6. Artemis does not localize in nucleolus.

With or without detergent pre-treatment, the localization of Artemis in HeLa cells was examined by IF. FBL acts as the nucleolus marker. Circled area indicates the nucleolus. Image bar: 10 μm.

Supplementary Figure S7

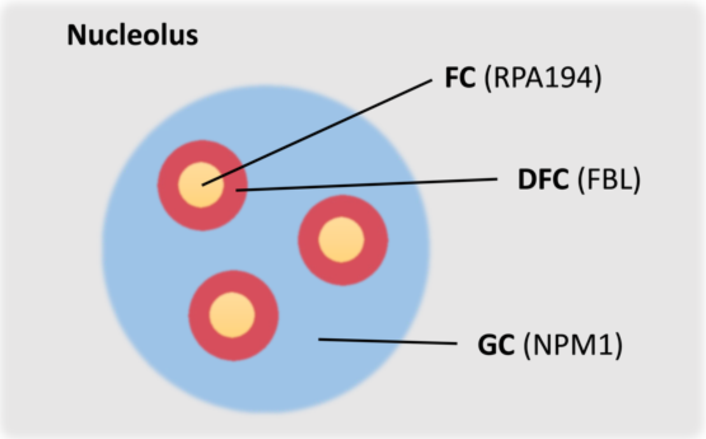

Supplementary Figure S7. Tripartite structure of nucleolus.

Schematic representation of the tripartite structure of nucleolus, formed by the fibrillar center (FC), the dense fibrillar component (DFC), and the granular component (GC). RPA194, FBL, and NPM1 are surrogate markers of FC, DFC, and GC components, respectively.

Supplementary Figure S8

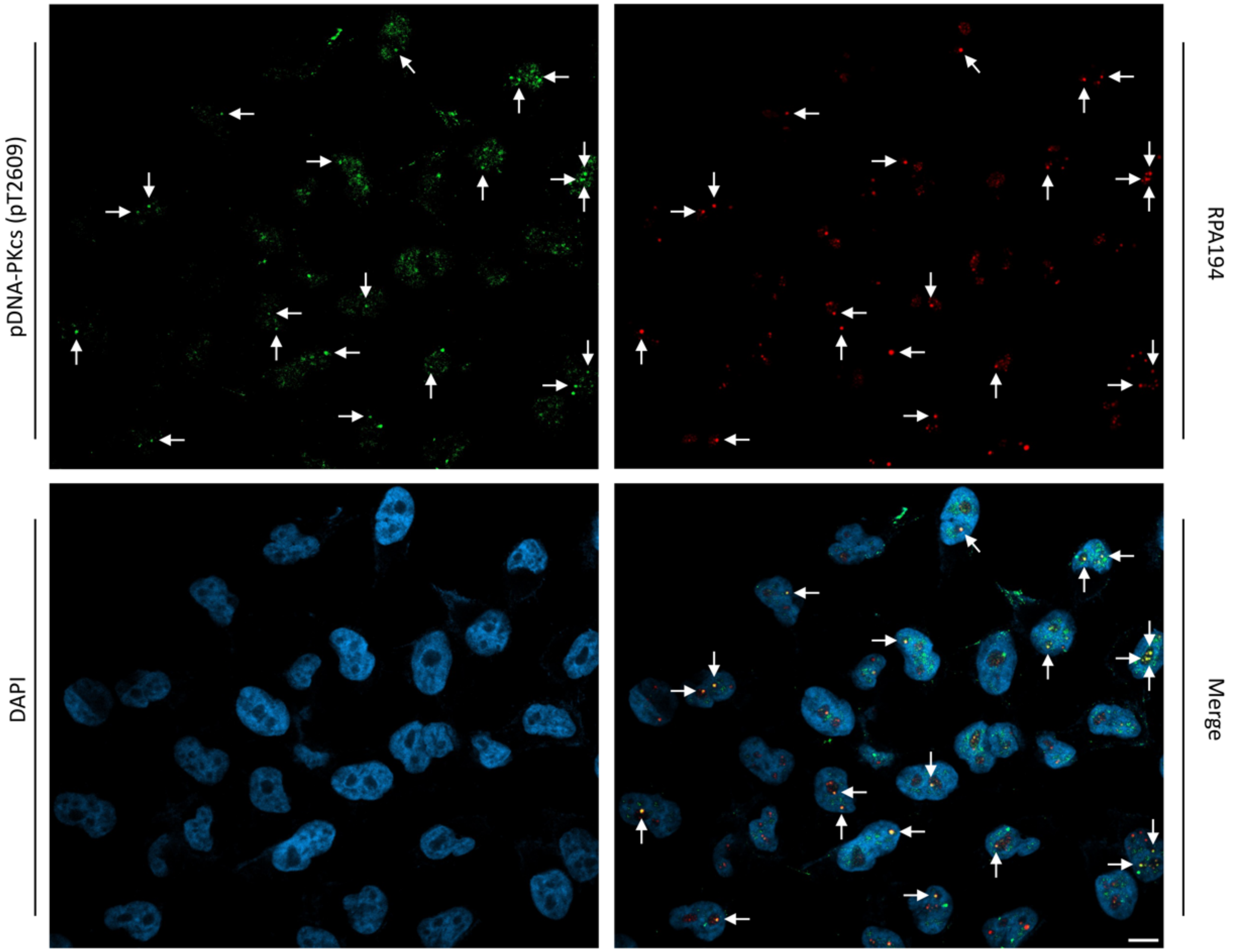

Supplementary Figure S8. The activated DNA-PK mainly colocalizes at the FC clusters.

Phosphorylated DNA-PKcs (pT2609) and RPA194 in HeLa cells were examined by IF. Arrows indicate the co-localization of pDNA-PKcs (pT2609) and RPA194. Image bar: 10 μm.

Supplementary Figure S9

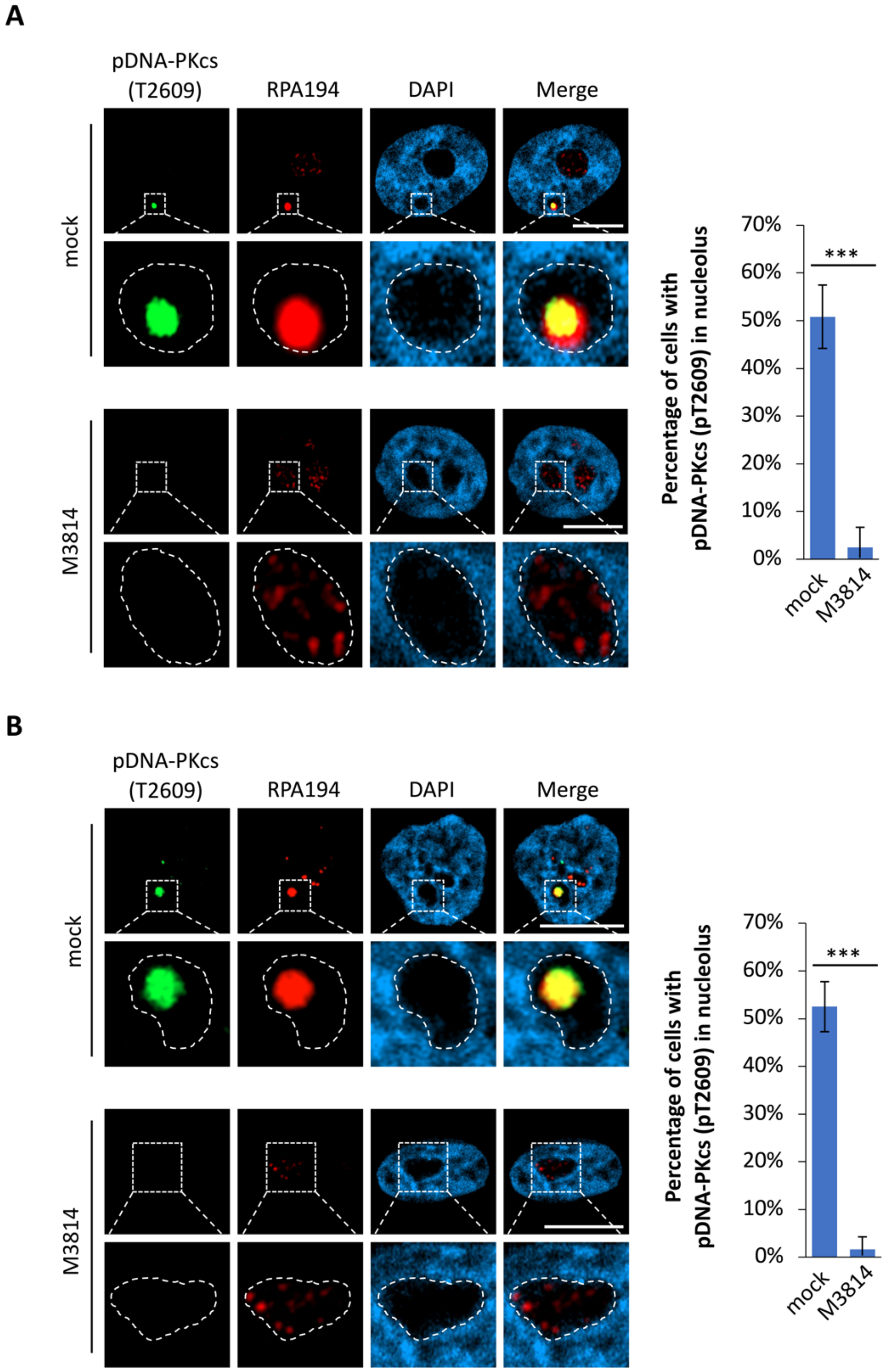

Supplementary Figure S9. DNA-PK-i treatment abrogates the activation of DNA-PK in nucleoli in *BRCA*-deficient tumor cells.

(**A, B**) UWB1 (**A**) or MDA-MB-436 (**B**) cells were pre-treated with or without M3814 (1 μM) for 24 hours. pT2609 of DNA-PKcs and RPA194 were examined by IF. Circled area indicates the nucleolus. The percentage of the cells with pDNA-PKcs (pT2609) staining in nucleolus was calculated. Image bar: 10 μm. ***$p < 0.001$.

Supplementary Figure S10

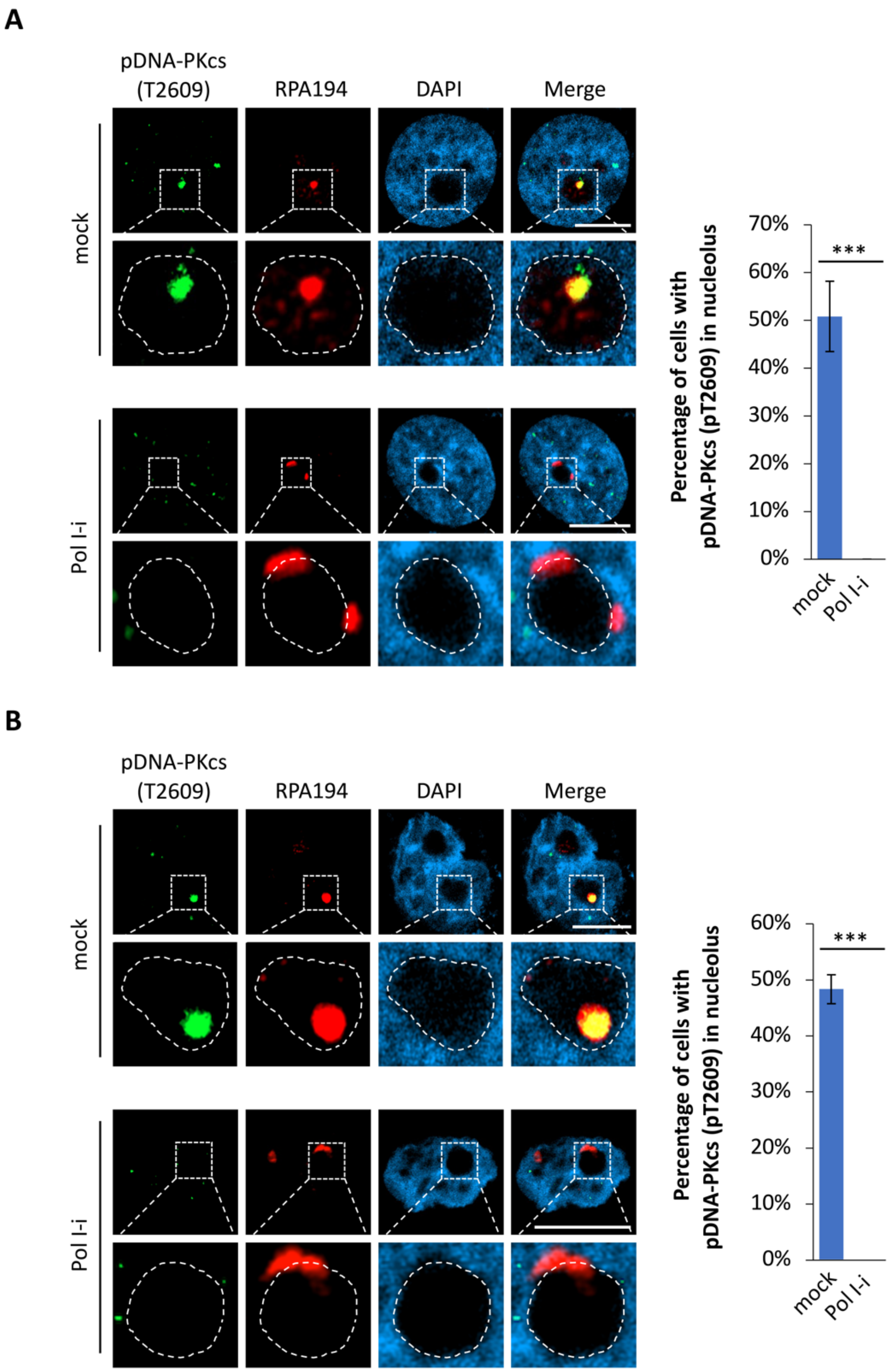

Supplementary Figure S10. Transient inhibition of RNA Pol I abolishes pDNA-PKcs (pT2609) in nucleoli in *BRCA*-deficient tumor cells.

(**A, B**) UWB1 (**A**) or MDA-MB-436 (**B**) cells were pre-treated with or without BMH-21 (1 μM) for 2 hours. pT2609 of DNA-PKcs and RPA194 were examined by IF. Circled area indicates the nucleolus. The percentage of the cells with pDNA-PKcs (pT2609) staining in nucleolus was calculated. Image bar: 10 μm. ***$p < 0.001$.

Supplementary Figure S11

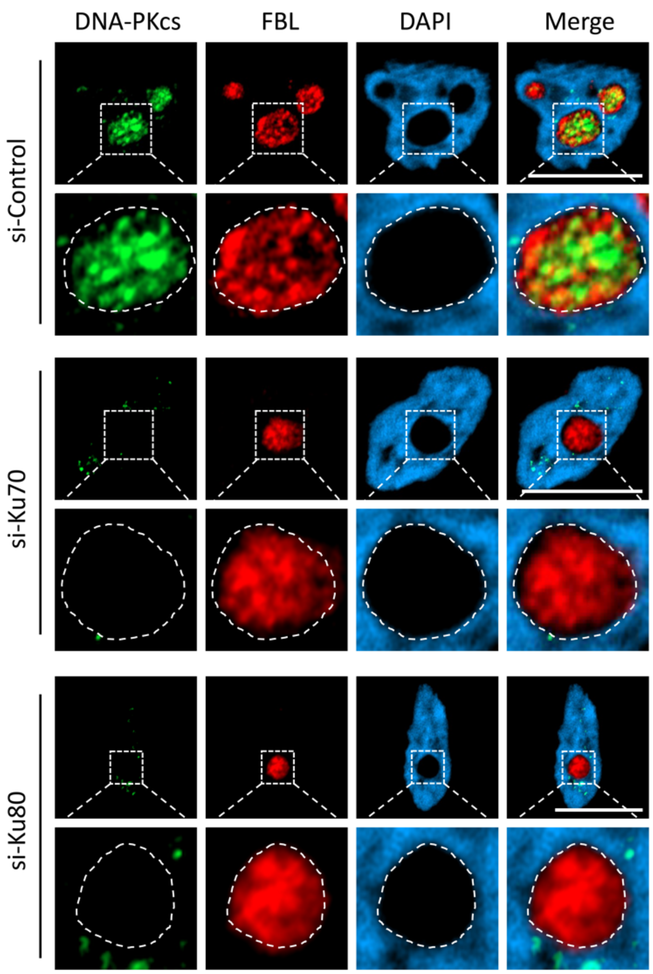

Supplementary Figure S11. DNA-PKcs nucleolar localization is dependent on Ku proteins.

Following knocking-down Ku proteins by siRNA in HeLa cells, the cells were pre-treated with detergent. DNA-PKcs and FBL were examined by IF. Circled area indicates the nucleolus. FBL acts as the nucleolus marker. Image bar: 10 μm.

Supplementary Figure S12

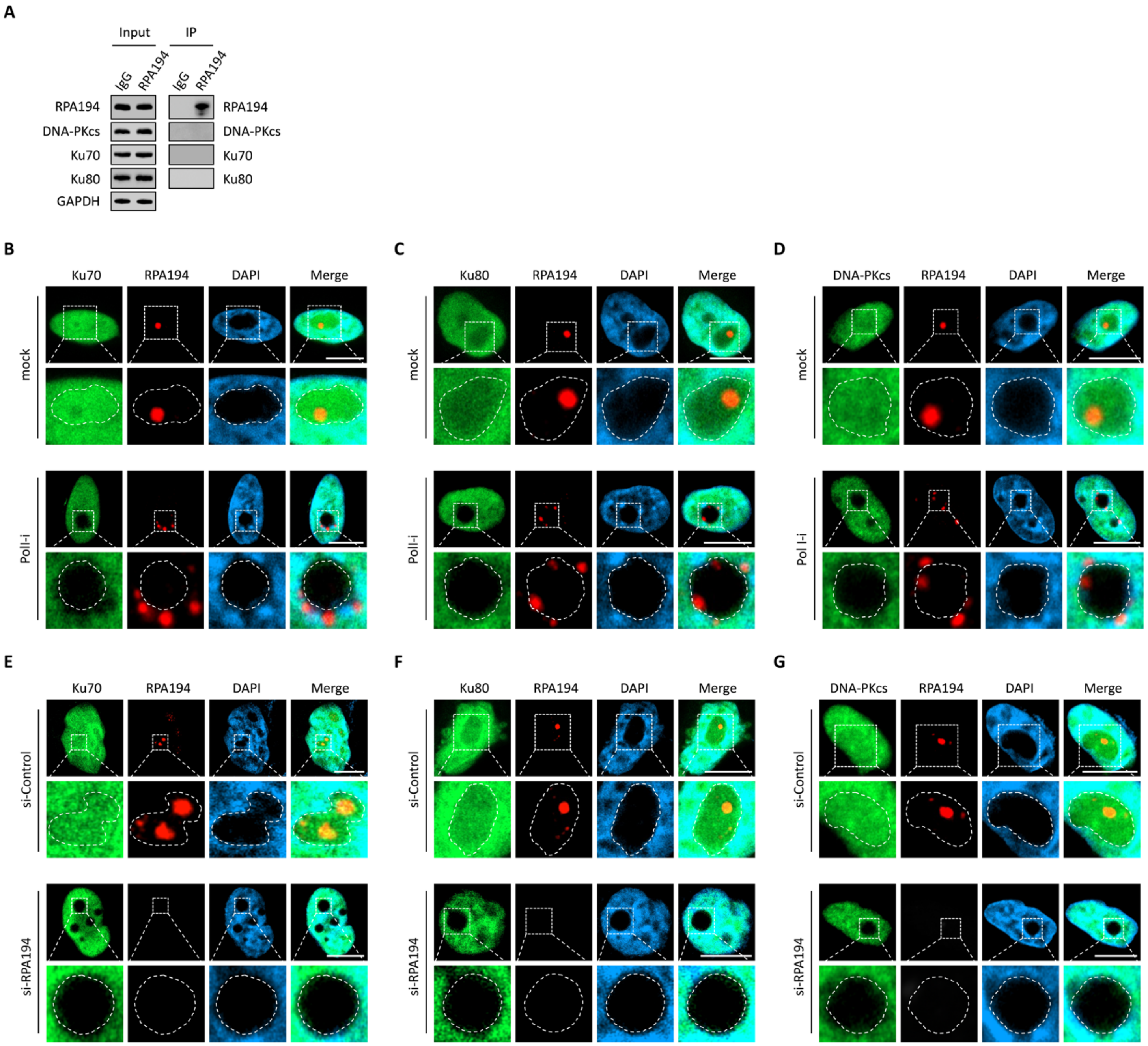

Supplementary Figure S12. DNA-PK nucleolar localization is dependent on Pol I-mediated rDNA transcription.

(**A**) DNA-PK does not interact with Pol I. The whole cell lysates from HeLa cells were subject to immunoprecipitation (anti-RPA194), and the immunoprecipitants were examined by Western blot. (**B-D**) DNA-PK nucleolar localization is dependent on Pol I-mediated rDNA transcription. HeLa cells were pre-treated with

or without BMH-21 (1 μM) for 2 hours. Ku70 (**B**) or Ku80 (**C**) or DNA-PKcs (**D**) and RPA194 were examined by IF. Circled area indicates the nucleolus. Image bar: 10 μm. (**E-G**) DNA-PK nucleolar localization is dependent on RPA194 protein. Following knocking-down RPA194 by siRNA in HeLa cells, Ku70 (**E**), Ku80 (**F**), or DNA-PKcs (**G**) and RPA194 were examined by IF. Circled area indicates the nucleolus. Image bar: 10 μm.

Supplementary Figure S13

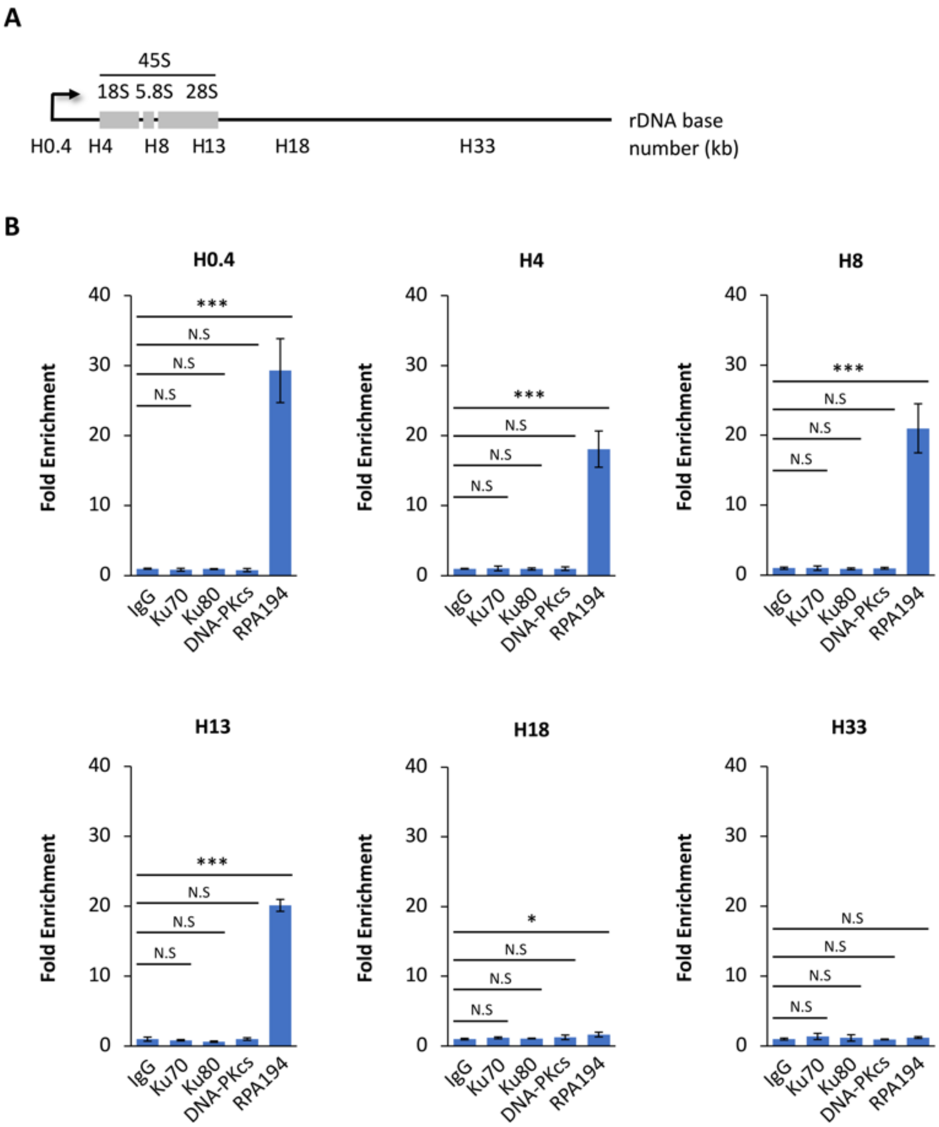

Supplementary Figure S13. DNA-PK does not bind to rDNA.

(**A**) The scheme of the human 45S rDNA unit shows the location of the amplicons used in this study. (**B**) The distribution of DNA-PK subunits and RPA194 (the catalytic subunit of RNA polymerase I) along the 45S rDNA repeat unit was determined by ChIP assays using HEK293T cells. *p < 0.1, **p < 0.01, ***p < 0.001.

Supplementary Figure S14

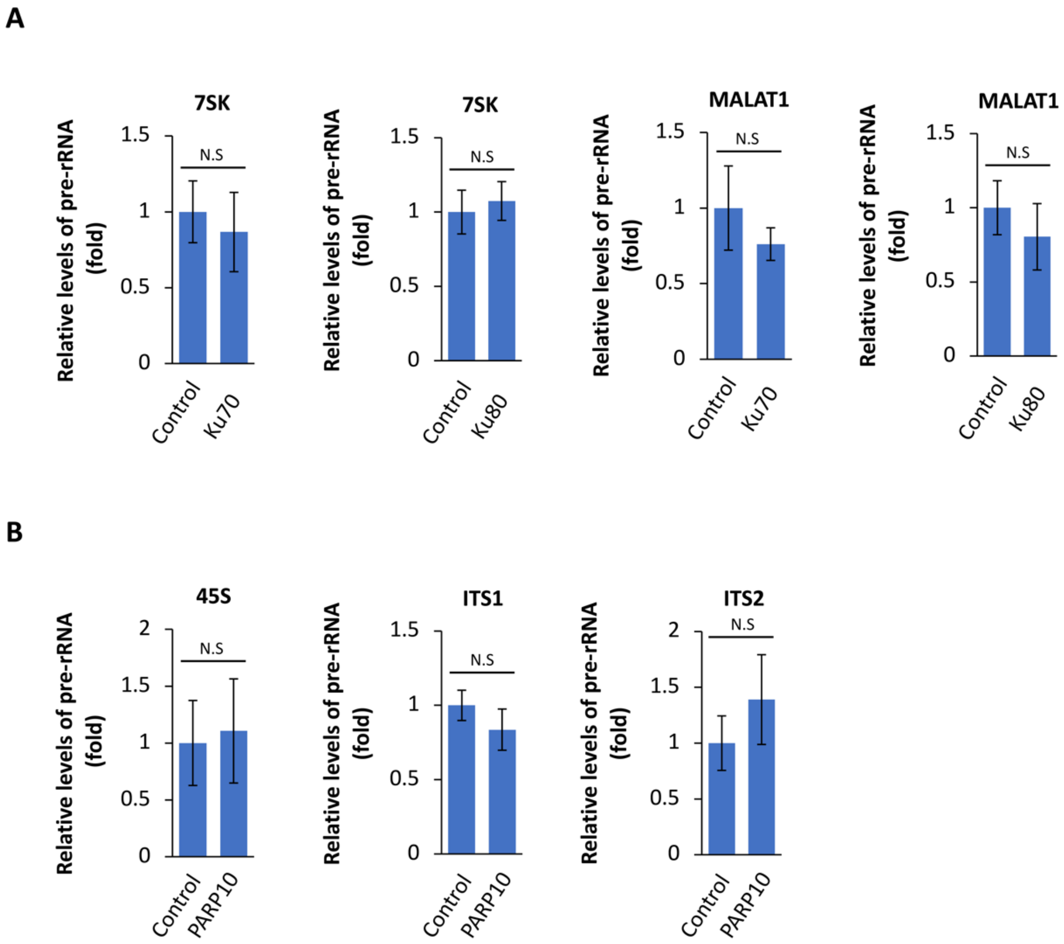

Supplementary Figure S14. The Ku complex does not associate with 7SK RNA or MALAT1 RNA.

(**A**) The Ku complex does not associate with 7SK or MALAT1. Endogenous Ku70 or Ku80 was immunoprecipitated from HEK293T cells, q-PCR was performed to examine the enrichment of 7SK or MALAT1. The irrelevant IgG acts as a control. (**B**) PARP10 does not associate with pre-rRNA. Endogenous PARP10 was immunoprecipitated from HEK293T cells, q-PCR was performed to examine the enrichment of pre-rRNA. The irrelevant IgG acts as a control.

Supplementary Figure S15

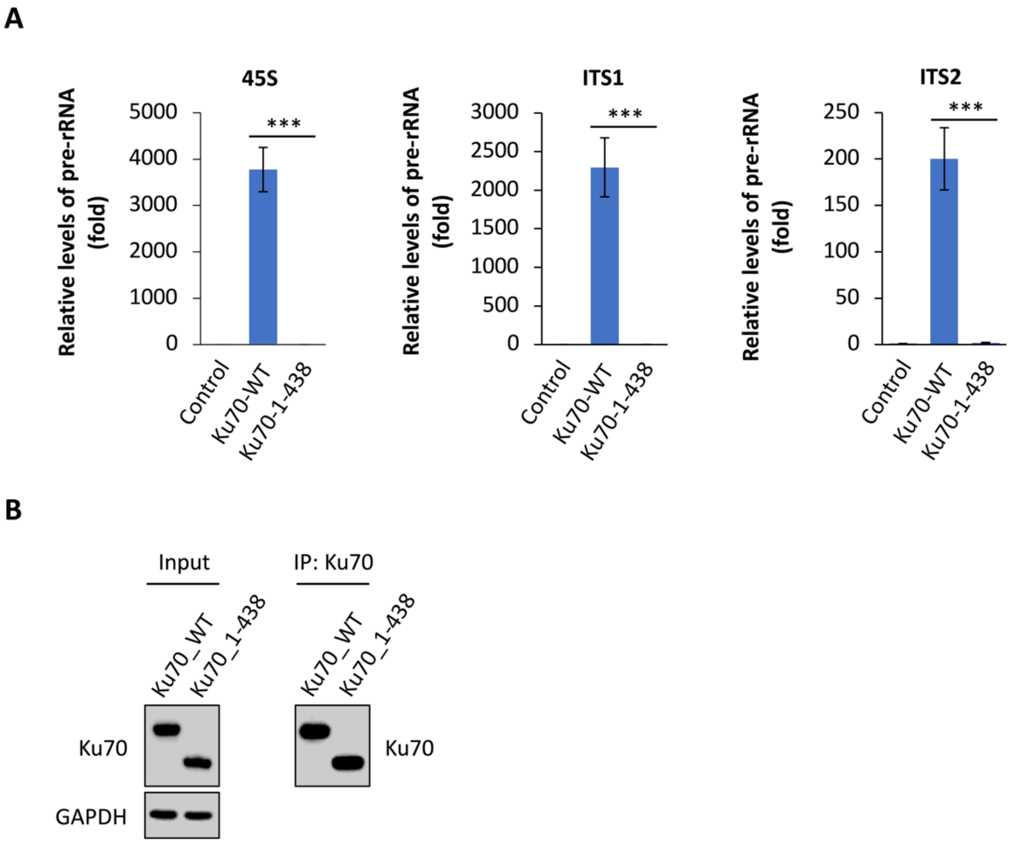

Supplementary Figure S15. The Ku70 mutant does not bind to pre-rRNA.

(**A, B**) Ku70-KO HEK293T cells were reconstituted with Ku70-WT or Ku70-1-438 mutant. Ku70-WT or Ku70-1-438 mutant was immunoprecipitated by anti-Ku70 antibody. RT-qPCR was performed to examine the enrichment of pre-rRNA (**A**). The whole cell lysates and the immunoprecipitants were examined by Western blot (**B**). ***p < 0.001.

Supplementary Figure S16

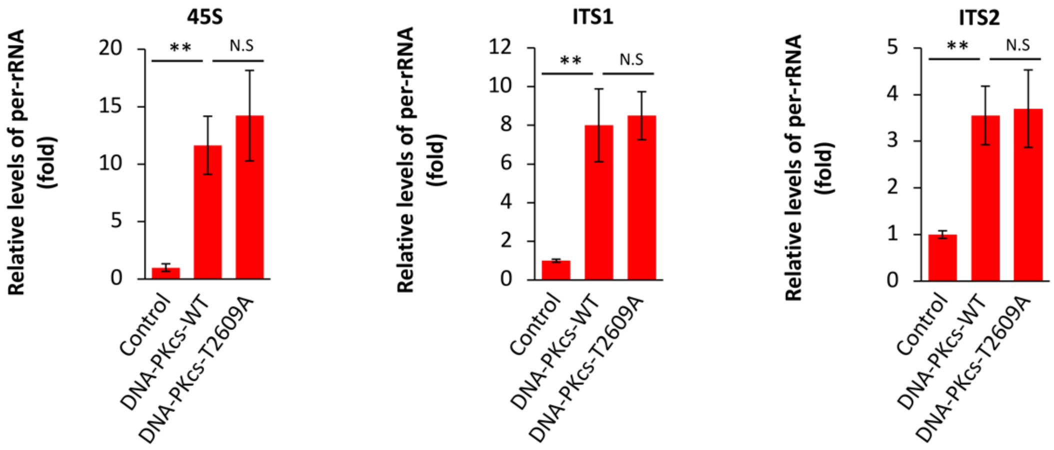

Supplementary Figure S16. DNA-PKcs associates with pre-rRNA.

DNA-PKcs-KO HEK293T cells were reconstituted with wild-type DNA-PKcs or DNA-PKcs-T2609A mutant. DNA-PKcs or DNA-PKcs-T2609A mutant was immunoprecipitated by anti-DNA-PKcs antibody, and RT-qPCR was performed to examine the enrichment of pre-rRNA. $**p < 0.01$.

Supplementary Figure S17

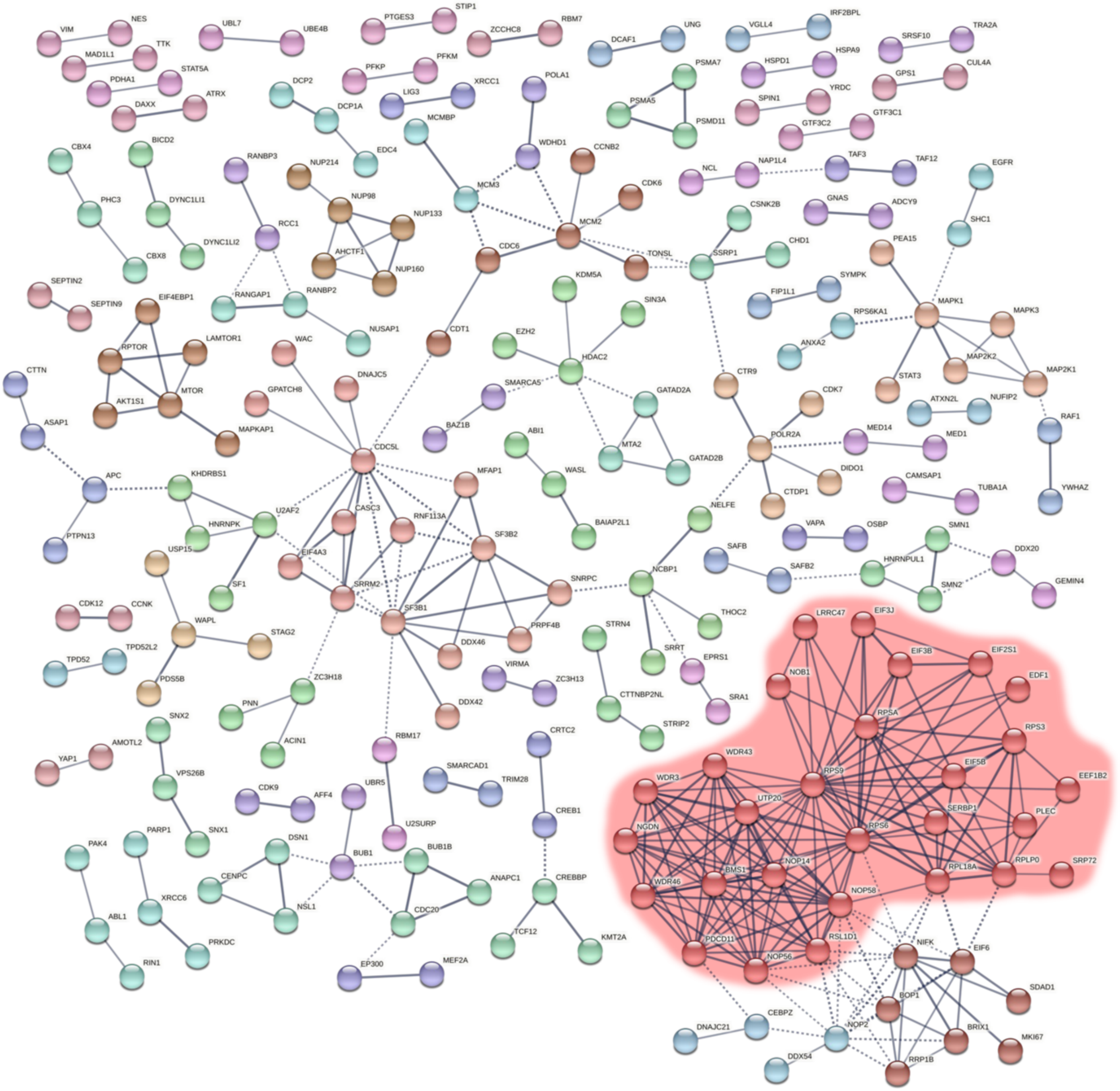

Supplementary Figure S17. Physical interactions among the substrates of DNA-PK.

Phosphoproteomic analyses show the network of physical connections among DNA-PK substrates as determined by String 12.0. The largest cluster is marked in red.

Supplementary Figure S18

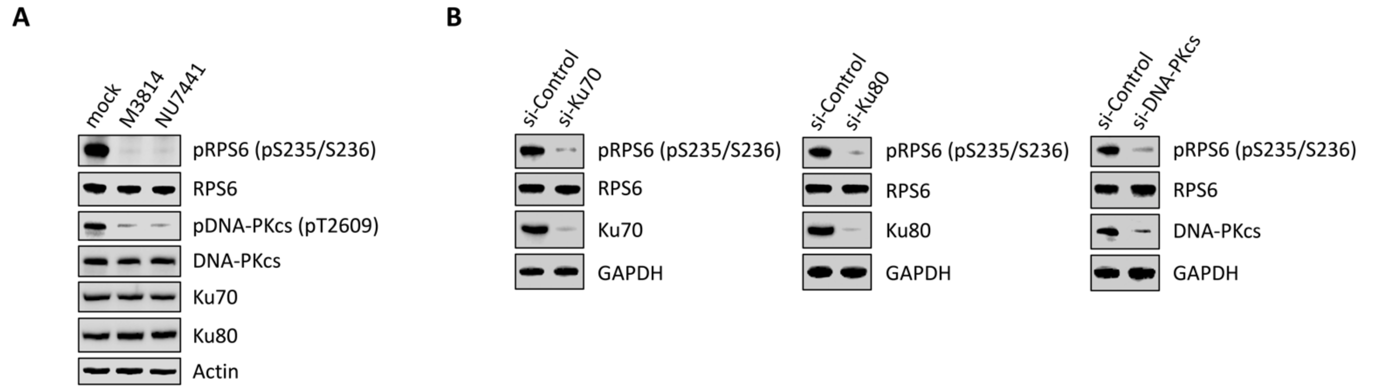

Supplementary Figure S18. DNA-PK-mediated the phosphorylation of RPS6 at S235/S236.

(**A**) DNA-PK inhibitor treatment abolishes the phosphorylation of RSP6 at S235/S236. HeLa cells were pre-treated with M3814 (1 μM) or NU7441 (1 μM) for 24 hours. pRPS6 (pS235/S236) was examined by Western blot. (**B**) Depletion of DNA-PK abolishes the phosphorylation of RPS6-S235/S236. Following knocking-down DNA-PK subunits by siRNA in HeLa cells, pRPS6 (pS235/S236) was examined by Western blot.

Supplementary Figure S19

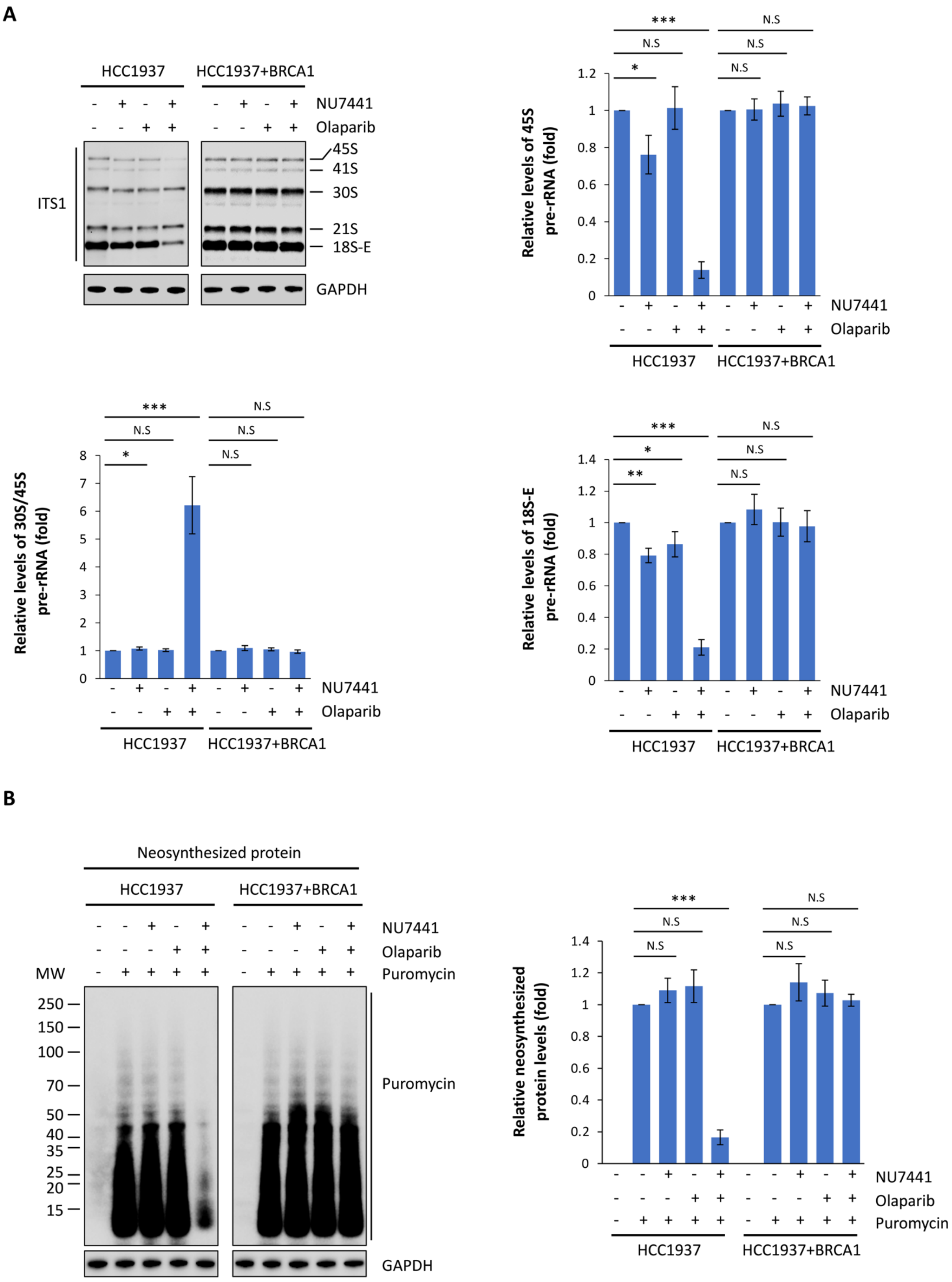

Supplementary Figure S19. DNA-PKcs-i acts together with PARP-i to suppress ribosome biogenesis and protein translation.

(**A**) NU7441 acts together with olaparib to suppress rRNA biogenesis. Northern blot analysis of pre-rRNA synthesis and processing on HCC1937 or HCC1937-BRCA1 cells with the pre-treatment of NU7441 (1 μM) and/or olaparib (1 μM) for 24 hours (left panel). Probes targeting ITS1 region were used. GAPDH was used as loading control. The relative levels of pre-rRNA were measured (right and lower panels). *p < 0.1, **p < 0.01, ***p < 0.001. (**B**) Protein synthesis is suppressed by the treatment of NU7441 and olaparib. HCC1937 or HCC1937-BRCA1 cells were pre-treated with NU7441 (1 μM) and/or olaparib (1 μM) for 24 hours. The relative neosynthesized protein levels were measured (right panel). ***p < 0.001.

Supplementary Figure S20

A

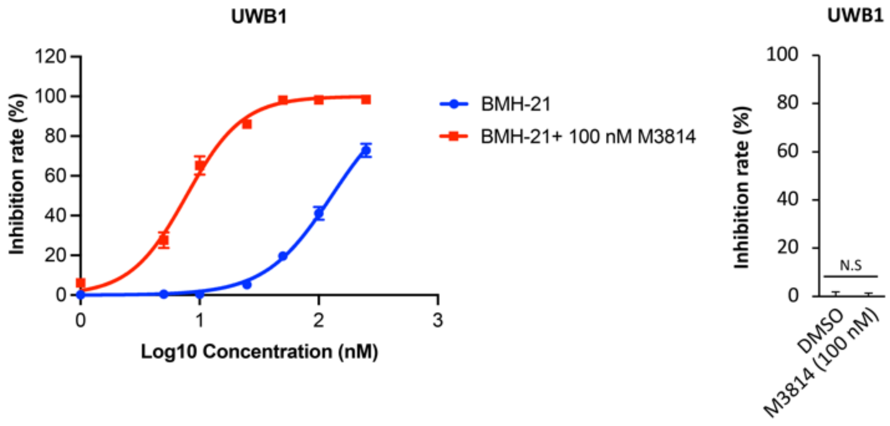

B

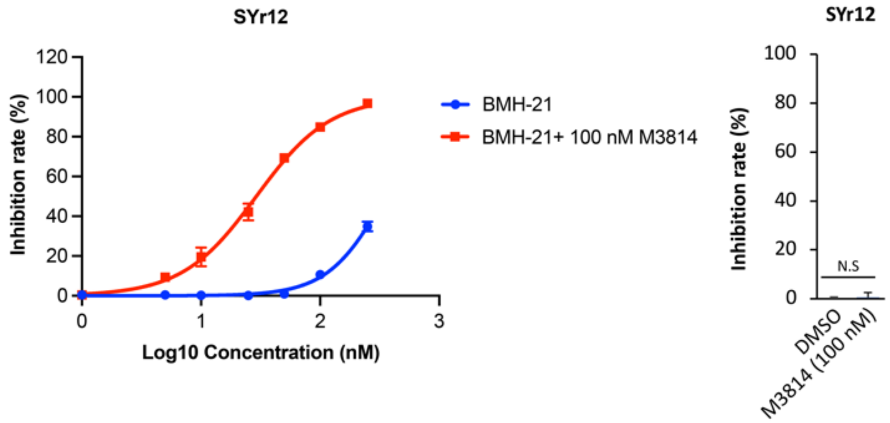

Supplementary Figure S20. DNA-PK-i and Pol I-i act together to suppress the growth of *BRCA*-deficient and PARP-i resistant tumor cells.

(**A**) DNA-PK-i and Pol I-i act together to suppress the growth of *BRCA*-deficient tumor cells. UWB1 cells were treated with the indicated dose of BMH-21, or BMH-21 together with 100 nM M3814 for 7 days. Cell growth was measured using CellTiter-Glo assays. The single arm of M3814 (100 nM) treatment in the cell viability assays is shown in the right panel. (**B**) DNA-PK-i and Pol I-i act together to suppress the growth of *BRCA*-deficient PARP-i resistant tumor cells. SYr12 cells were treated with the indicated dose of BMH-21, or BMH-21 together with 100 nM M3814 together for 7 days. Cell growth was measured using CellTiter-Glo assays. The single arm

of M3814 (100 nM) treatment in the cell viability assays is shown in the right panel. Average cell growth suppression is presented as mean ± SD.

Supplementary Table S1. The full PAR-CLIP analysis results.

Supplementary Table S2. List of DNA-PK phosphorylation sites on substrates.

Supplementary Table S3. List of DNA-PK phosphoproteins.
